# Optimal Medical Therapy for Heart Failure and Integrated Care in Patients With Atrial Fibrillation: A Report From the ESC‐EHRA EORP Atrial Fibrillation Long‐Term General Registry

**DOI:** 10.1161/JAHA.123.030499

**Published:** 2024-12-20

**Authors:** Niccolò Bonini, Marco Proietti, Giulio Francesco Romiti, Marco Vitolo, Ameenathul Mazaya Fawzy, Wern Yew Ding, Jacopo Francesco Imberti, Laurent Fauchier, Francisco Marin, Michael Nabauer, Gheorghe Andrei Dan, Tatjana S. Potpara, Giuseppe Boriani, Gregory Y. H. Lip, the ESC‐EHRA EORP‐AF General Long‐Term Registry Investigators

**Affiliations:** ^1^ Liverpool Centre for Cardiovascular Science at University of Liverpool, Liverpool John Moores University and Liverpool Heart & Chest Hospital Liverpool United Kingdom; ^2^ Cardiology Division, Department of Biomedical, Metabolic and Neural Sciences University of Modena and Reggio Emilia, Policlinico di Modena Modena Italy; ^3^ Department of Clinical Sciences and Community Health University of Milan Milan Italy; ^4^ Division of Subacute Care IRCCS Istituti Clinici Scientifici Maugeri Milan Italy; ^5^ Department of Translational and Precision Medicine Sapienza—University of Rome Italy; ^6^ Clinical and Experimental Medicine PhD Program University of Modena and Reggio Emilia Modena Italy; ^7^ Service de Cardiologie Centre Hospitalier Universitaire Trousseau Tours France; ^8^ Department of Cardiology Hospital Universitario Virgen de la Arrixaca, IMIB‐Arrixaca, University of Murcia, CIBER‐CV Murcia Spain; ^9^ Department of Cardiology Ludwig‐Maximilians‐University Munich Germany; ^10^ University of Medicine, ‘Carol Davila’ Colentina University Hospital Bucharest Romania; ^11^ School of Medicine University of Belgrade Serbia; ^12^ Intensive Arrhythmia Care, Cardiology Clinic Clinical Center of Serbia Belgrade Serbia; ^13^ Danish Center for Health Services Research, Department of Clinical Medicine Aalborg University Aalborg Denmark

**Keywords:** atrial fibrillation, heart failure, integrated care, outcomes, Atrial Fibrillation

## Abstract

**Background:**

Heart failure (HF) often occurs in patients with atrial fibrillation (AF), with a major impact on prognosis. Few data are available on the effect of integrated treatment strategies to improve prognosis in patients with AF. We aimed to evaluate the association between HF (according to left ventricular ejection fraction [LVEF]), HF optimal medical therapy and adherence to the Atrial Fibrillation Better Care pathway, and major outcomes in patients with AF.

**Methods and Results:**

From the ESC‐EHRA EORP‐AF (European Society of Cardiology–European Heart Rhythm Association EURObservational Research Programme in Atrial Fibrillation) General Long‐Term Registry, we evaluated patients with HF, categorized according to LVEF (HF with reduced ejection fraction, HF with mildly reduced ejection fraction, HF with preserved ejection fraction). Optimal medical therapy for HF was guidelines‐defined. The primary end point was the composite of all‐cause death and major adverse cardiovascular events. From the original cohort, 9373 (84.5%) patients were included in this analysis (median age, 71 [interquartile range, 62–77] years; 39.9% women). Compared with no HF, all HF categories were associated with an increased risk of the primary composite outcome, with highest figures observed for HF with reduced ejection fraction (hazard ratio [HR], 2.36 [95% CI, 2.00–2.78]). The risk was reduced in patients with AF and HF adherent to optimal medical therapy (HR, 0.83 [95% CI, 0.70–0.98]), as well as in those adherents to the Atrial Fibrillation Better Care pathway (HR, 0.65 [95% CI, 0.48–0.88]). The effect of Atrial Fibrillation Better Care pathway was consistent across the spectrum of LVEF.

**Conclusions:**

Patients with AF and HF have a high risk of major adverse events, and this risk is inversely associated with LVEF. Atrial Fibrillation Better Care pathway adherent management is associated with improved clinical outcomes in patients with HF, across the spectrum of LVEF.

**Nonstandard Abbreviations and Acronyms**
ABC: Atrial Fibrillation Better Care
CABANA: Catheter Ablation Versus Anti‐arrhythmic Drug Therapy for Atrial Fibrillation
CASTLE‐AF: Catheter Ablation Versus Standard Conventional Therapy in Patients with Left Ventricular Dysfunction and Atrial Fibrillation
EHRA: European Heart Rhythm Association
ESC‐EHRA EORP‐AF: European Society of Cardiology–European Heart Rhythm Association EURObservational Research Programme in Atrial Fibrillation
HFpEF: heart failure with preserved ejection fraction
HFmrEF: heart failure with mildly reduced ejection fraction
HFrEF: heart failure with reduced ejection fraction
MACEs: major adverse cardiovascular events
OAC: oral anticoagulant
OMT: optimal medical therapy

**Clinical Perspective**

**What Is New?**

- In patients with atrial fibrillation, concomitant presence of heart failure (HF) is associated with a higher risk of major adverse outcomes, with a continuous, nonlinear association between left ventricular ejection fraction and risk of outcomes in both HF and overall cohort of patients with atrial fibrillation.
- Optimal medical therapy for HF is associated with a reduction in risk of major adverse outcomes, particularly in those with HF with reduced ejection fraction, while the application of the Atrial Fibrillation Better Care pathway in such patients showed an association with a greater reduction of risk, irrespective of left ventricular ejection fraction.

**What Are the Clinical Implications?**

- In patients with atrial fibrillation and HF, implementation of optimal medical therapy in the context of an integrated and holistic care through the Atrial Fibrillation Better Care pathway would be likely able to reduce the high risk of major adverse outcomes demonstrated in these patients.

Heart failure (HF) commonly occurs in patients with atrial fibrillation (AF) and vice versa.^1^ Therefore, patients suffering from both AF and HF have a much higher risk of clinical adverse outcomes than patients with only 1 condition.^1^ Due to the clinical complexity of these patients, an intensive and structured therapeutic management pathway is one of the possible solutions to improve their prognosis.^2^

In 2017, the Atrial Fibrillation Better Care (ABC) pathway was proposed as a strategy to streamline and integrate holistic care for patients with AF, and it was subsequently associated with a lower risk of major outcomes^3^, ^4^ and included in international guidelines on the management of AF.^5^, ^6^ The ABC pathway is based on 3 main criteria: “A,” avoid stroke, through appropriate anticoagulation therapy; “B,” better symptom management, with rate or rhythm control approaches; and “C,” cardiovascular and comorbidity risk optimization.^3^

Indeed, patients with HF are usually complex and require comprehensive care, whereby the cornerstone of management is so‐called guidelines‐direct optimal medical therapy (OMT), particularly for patients with HF with reduced ejection fraction (HFrEF).^7^ In patients with AF and HF, the adherence to the ABC pathway is inevitably influenced by adherence to the HF OMT, given the obvious importance in determining adherence to the “C” criterion. Furthermore, patients with HF with preserved ejection fraction (HFpEF) are burdened by a high risk of major adverse outcomes,^8^ and AF exerts a detrimental role in these patients.^9^

To date, there are limited data on the risk of major adverse outcomes in patients with AF and HF as a function of left ventricular ejection fraction (LVEF) levels and how adherence to the ABC pathway and to OMT may influence this risk across the HF spectrum.

The aims of this study are (1) to describe the characteristics of patients with HF in a large contemporary European cohort of patients with AF; (2) to assess the impact of HF on the risk of major adverse outcomes in patients with AF, across the whole spectrum of LVEF; and (3) to analyze the impact and interplay of adherence to OMT and ABC pathway on the risk of major adverse outcomes. To achieve the study objectives, we performed an analysis of the ESC‐EHRA EORP‐AF (European Society of Cardiology–European Heart Rhythm Association EURObservational Research Programme in Atrial Fibrillation) Long‐Term General Registry.

## METHODS

The ESC‐EHRA EORP‐AF General Long‐Term Registry is a prospective, observational, multicenter registry, held by the European Society of Cardiology and endorsed by the European Heart Rhythm Association. The study enrolled consecutive AF inpatients and outpatients in 250 cardiology practices, across 27 countries. Details of the study design, baseline characteristics, and follow‐up are reported elsewhere.^10^, ^11^

All patients enrolled had documented AF within 12 months before enrollment, were aged ≥18 years, and provided written informed consent. Enrollment was undertaken from October 2013 to September 2016, with planned 1‐ and 2‐year follow‐up.

Patient data were obtained after the signing of a written informed consent by each patient, following the approval of study protocol by an institutional review board/ethics committee. The study was first approved by the National Coordinators' main institutions and subsequently was authorized by each peripheral site under the responsibility of the lead contact and study team according to the specific national and local regulation. Any details regarding approval numbers for the study protocol regarding any specific site could be obtained from the corresponding authors upon reasonable request. The study was performed according to the European Union Note for Guidance on Good Clinical Practice CPMP/ECH/135/95 and the Declaration of Helsinki.

For the purposes of this analysis, we considered all patients with available data regarding the presence or absence of HF, as per the clinical evaluation and medical history analysis made by the investigators at the moment of the study enrollment, LVEF evaluation, and occurrence of major adverse outcomes during the follow‐up period.

### Study Cohort and Definitions

For the purposes of this analysis, we considered all patients with available data on HF status, LVEF, and occurrence of major adverse outcomes during the follow‐up period. According to the European Society of Cardiology guidelines on HF management at the time the data were collected,^7^ for the primary analysis patients with HF were initially categorized by LVEF as follows: (1) patients with reduced LVEF (HFrEF); (2) patients with mildly reduced LVEF (HFmrEF); and (3) patients with preserved LVEF (HFpEF). Secondarily, to perform various subgroup analyses and not to excessively reduce the sample of each group examined, we adopted a simplified categorization: (1) patients with HF with reduced LVEF (≤40%); (2) patients with HF without reduced LVEF (>40%).

Symptomatic AF status was defined according to EHRA score, while thromboembolic and bleeding risks were assessed according to CHA_2_DS_2_‐VASc and HAS‐BLED scores, computed according to the original schemes.^12^, ^13^ We defined high thromboembolic risk when CHA_2_DS_2_‐VASc was ≥2 in men and ≥3 in women, and high bleeding risk when HAS‐BLED was ≥3. Type of AF was classified according to the current European guidelines^6^ as (1) first detected AF, (2) paroxysmal AF, (3) persistent AF, (4) long‐standing persistent AF, and (5) permanent AF.

Symptomatic status related to HF was defined according to the New York Heart Association classification. OMT for HF was defined as the complementary and contemporary use of any angiotensin‐converting enzyme inhibitor or an angiotensin receptor blocker and β blockers or mineralocorticoid receptor antagonist, thus a minimum of 2 medications among angiotensin‐converting enzyme inhibitor/angiotensin receptor blocker and β blocker/mineralocorticoid receptor antagonist, according to European Society of Cardiology 2012 HF management guidelines, which were valid throughout the study period.^14^

Adherence to the ABC pathway was evaluated at baseline and defined as per previously published study^15^ according to 3 criteria^3^:

- “A” criterion: Patients were considered “adherent” to the “A” criterion if properly prescribed with an OAC according to their thromboembolic risk. Specifically, we considered adherent men with CHA_2_DS_2_‐VASc score ≥1 and women with CHA_2_DS_2_‐VASc score ≥2, treated with either vitamin K antagonist (with a time in therapeutic range ≥70%) or a non–vitamin K antagonist OAC; patients not receiving an OAC and with low thromboembolic risk (ie, CHA_2_DS_2_‐VASc score of 0 in men or 1 in women) were also considered adherent.
- “B” criterion: As this criterion refers to the *actual* symptom control, rather than the *attempt*, we considered “adherent” those patients with a European Heart Rhythm Association score of I (no symptoms) or II (mild symptoms) at baseline.
- “C” criterion: For this criterion, we considered the comorbidities most frequently found in patients with AF: hypertension, coronary artery disease, peripheral artery disease, HF, previous stroke/transient ischemic attack, and diabetes. Any patient with ≥1 of these conditions and treated according to optimal medical treatment (defined according to the current clinical guidelines) was considered adherent to this criterion. Optimal treatment was defined as follows: (1) hypertension: if blood pressure at baseline was ≤140/90 mm Hg; (2) coronary artery disease: treatment with angiotensin‐converting enzyme inhibitors, β blockers, and statins; (3) peripheral artery disease: treatment with statins; (4) previous stroke/transient ischemic attack: treatment with statins; (5) HF: treatment with angiotensin‐converting enzyme inhibitors or angiotensin receptor blockers, and β blockers; and (6) diabetes: treatment with insulin or oral antidiabetics. Patients with ≥2 of the above conditions needed to be optimally treated for all to be considered adherent to the “C” criterion.

Patients who met all 3 criteria were considered adherent to the ABC pathway; otherwise, they were considered ABC nonadherent.^8^

### Major Adverse Outcomes

For this study, we considered as the primary outcome the composite of all‐cause death and major adverse cardiovascular events (MACEs) comprising any thromboembolic event, any acute coronary syndrome and cardiovascular death. As exploratory secondary outcomes, we also evaluated both all‐cause death and MACEs as separate outcomes, as well as the occurrence of HF worsening/hospitalization in patients with HF.

### Statistical Analysis

All continuous variables were reported as median and interquartile range (IQR). Between‐group comparisons were made using nonparametric tests, Mann–Whitney U test (comparison between 2 groups), or Kruskal–Wallis ANOVA test (comparison between multiple groups), where appropriate. Categorical variables were reported as counts and percentages. Among‐group comparisons were made using the χ^2^ test.

Differences in survival according to subgroups examined for the main clinical outcome (composite outcome) were analyzed with the log‐rank test, and Kaplan–Meier curves were reported accordingly. Cox regression analyses were performed to evaluate the association between exposures and risk of outcomes and were adjusted with the following covariates: age, sex, hypertension, diabetes, coronary artery disease, peripheral artery disease, previous thromboembolic events, history of malignancy (current or prior in remission), chronic kidney disease, chronic obstructive pulmonary disease, and type of AF; with additional adjustment for the use of OAC for models analyzing the association of HF with major outcomes, and adjustment for HF subtype (ie, HFrEF, HFmrEF, and HFpEF) for model analyzing the association of adherence to OMT and the ABC pathway on the risk of major outcomes. Results were reported as hazard ratio (HR) and 95% CIs; proportional hazard assumption was assessed through visual inspection of Schoenfeld residuals, with no critical violation observed. We modeled regression curves to analyze the association between continuous values of ejection fraction (EF) and risk of the main clinical outcome, adjusted for the same covariates listed above. In case of nonlinearity, restricted cubic splines with 3 knots at the 25th, 50th, and 75th percentiles were used to model the regressions, with reference value placed at LVEF=55% for all the analyses. The Wald statistical test for nonlinearity was also performed and reported. A 2‐sided *P*<0.05 was considered statistically significant. All analyses were performed using SPSS statistical software version 27.0.1.0 (IBM, Armonk, NY) for Mac OS, and R 4.4.0 (R Core Team, Vienna, Austria) for Windows.

## RESULTS

Among the original 11 096 patients enrolled in the ESC‐EHRA EORP‐AF registry, 9373 (84.5%) were available for this analysis. Median age was 71 [IQR, 62–77] years, 3740 (39.9%) were women; median CHA_2_DS_2_‐VASc and HAS‐BLED were, respectively, 3 [IQR, 2–4] and 1 [IQR, 1–2]. Overall, 3420 (36.5%) patients had HF; of these, 1662 (17.7%) had HFpEF, 523 (5.6%) HFmrEF, and 1235 (13.2%) HFrEF. According to the simplified categorization, 2185 (23.3%) had HF without reduced LVEF, and 1235 (13.2%) had HF with reduced LVEF.

Baseline characteristics according to presence and class of HF are reported in Table 1 and Table S1, while baseline characteristics according to the simplified categorization are shown in Table S2. Patients without HF were younger, while patients with HFpEF were the oldest. The prevalence of female sex was higher in patients with HFpEF and lower in patients with HFrEF. Permanent AF was more prevalent among patients with HFrEF, while both first diagnosed and paroxysmal were more likely prevalent in patients without HF. Patients with HFrEF were more likely symptomatic according to both the New York Heart Association class and European Heart Rhythm Association score. Regarding comorbidities and risk factors, most were more prevalent in patients with HF and specifically among patients with HFrEF, except for hypertension, lipid disorders, hemorrhagic events, malignancy, and hyperthyroidism being more prevalent in patients with HFpEF, with chronic obstructive pulmonary disease and obstructive sleep apnea syndrome more prevalent in patients with HFmrEF. Both CHA_2_DS_2_‐VASc and HAS‐BLED were significantly lower in patients with AF without HF.

**Table 1:** Baseline Characteristics of the Study Population According to Presence and Type of Heart Failure

Variables, n (%) N=9373	No HF n=5953	HFpEF n=1662	HFmrEF n=523	HFrEF n=1235	*P* value
Age, y, median (IQR)	69 (61–76)	74 (66–79)	73 (65–79)	71 (63–78)	<0.001
Female sex	2307/5953 (38.8)	885/1662 (53.2)	190/523 (36.3)	358/1235 (29.0)	<0.001
Body mass index, kg/m^2^, median (IQR)	27.7 (24.9–31.1)	27.7 (24.8–31.6)	27.6 (24.8–31.6)	27.1 (24.2–30.6)	<0.001
Region of enrollment					
Western Europe	2225/5953 (37.4)	542/1662 (32.6)	178/523 (34.0)	268/1235 (21.7)	<0.001
Southern Europe	2153/5953 (36.2)	495/1662 (29.8)	189/523 (36.1)	436/1235 (35.3)	
Northern Europe	1020/5953 (17.1)	82/1662 (4.9)	22/523 (4.2)	136/1235 (11.0)	
Eastern Europe	555/5953 (9.3)	543/1662 (32.7)	134/523 (25.6)	395/1235 (32.0)	
AF type	5826/5953 (97.8)	1651/1662 (99.3)	519/523 (99.2)	1220/1225 (98.8)	<0.001
First diagnosed	1027/5826 (17.6)	181/1651 (11.0)	77/519 (14.8)	178/1220 (14.6)	
Paroxysmal	1763/5826 (30.3)	394/1651 (23.9)	72/519 (13.9)	156/1220 (12.8)	
Persistent	1278/5826 (21.9)	267/1651 (16.2)	82/519 (15.8)	208/1220 (17.0)	
Long‐standing persistent	242/5826 (4.2)	86/1651 (5.2)	41/519 (7.9)	55/1220 (4.5)	
Permanent	1516/5826 (26.0)	723/1651 (43.8)	247/519 (47.6)	623/1220 (51.1)	
Hypertension	3486/5916 (58.9)	1159/1654 (70.1)	357/518 (68.9)	729/1224 (59.6)	<0.001
Diabetes	1116/5919 (18.9)	476/1656 (28.7)	170/522 (32.6)	376/1224 (30.7)	<0.001
Lipid disorder	2201/5723 (38.5)	762/1577 (48.3)	240/508 (47.2)	533/1186 (44.9)	<0.001
Smoking (current)	535/5477 (9.8)	98/1580 (6.2)	43/503 (8.5)	136/1138 (12.0)	<0.001
Alcohol (any intake)	2046/5173 (39.5)	421/1535 (27.4)	149/487 (30.5)	383/1091 (35.1)	<0.001
New York Heart Association class	…				<0.001
I	…	293/1661 (17.6)	80/523 (15.3)	110/1234 (8.9)	
II	…	895/1661 (53.9)	267/523 (51.1)	500/1234 (40.5)	
III	…	413/1661 (24.8)	151/523 (28.9)	501/1234 (40.6)	
IV	…	60/1661 (3.6)	25/523 (4.8)	123/1234 (10.0)	
EHRA score III–IV	974/5953 (16.4)	335/1662 (20.2)	134/523 (25.6)	357/1235 (28.9)	<0.001
Coronary artery disease	1222/5810 (21.0)	535/1501 (35.6)	207/475 (43.6)	562/1128 (49.8)	<0.001
Previous myocardial infarction	492/1222 (40.3)	178/535 (33.3)	97/207 (46.9)	347/562 (61.7)	<0.001
Previous angina	343/1222 (28.1)	223/535 (41.7)	66/207 (31.9)	159/562 (28.3)	<0.001
Previous PCI	574/1222 (47.0)	186/535 (34.8)	74/207 (35.7)	234/562 (41.6)	<0.001
Previous CABG	245/1222 (20.0)	78/535 (14.6)	48/207 (23.2)	112/562 (19.9)	0.016
LVEF, %, median (IQR)	60 (53–64)	58 (54–64)	45 (45–46)	31 (25–38)	<0.001
LVH	1173/4816 (24.4)	587/1548 (37.9)	208/490 (42.4)	280/1141 (24.5)	<0.001
Valvular alterations	2404/5848 (41.1)	1095/1651 (66.3)	318/515 (61.7)	893/1226 (72.8)	<0.001
Any cardiomyopathy	380/5953 (6.4)	229/1662 (13.8)	158/523 (30.2)	686/1235 (55.5)	<0.001
Dilated	129/380 (33.9)	70/229 (30.6)	93/158 (58.9)	513/686 (74.8)	0.000
Hypertrophic	124/380 (32.7)	93/229 (40.7)	22/158 (13.9)	51/686 (7.5)	<0.001
Previous thromboembolic events	636/5927 (10.7)	210/1639 (12.8)	67/520 (12.9)	167/1210 (13.8)	0.004
Hemorrhagic events	241/5922 (4.1)	129/1643 (7.9)	38/519 (7.3)	80/1213 (6.6)	<0.001
Peripheral vascular disease	325/5887 (5.5)	205/1614 (12.7)	54/515 (10.5)	153/1188 (12.9)	<0.001
Liver disease	92/5934 (1.6)	63/1648 (3.8)	20/518 (3.9)	77/1229 (6.3)	<0.001
COPD	381/5913 (6.4)	213/1646 (12.9)	74/520 (14.2)	151/1225 (12.3)	<0.001
Dementia	37/5943 (0.6)	36/1654 (2.2)	6/522 (1.1)	38/1224 (3.1)	<0.001
Anemia	176/5942 (3.0)	143/1656 (8.6)	44/520 (8.5)	133/1232 (10.8)	<0.001
Malignancy	471/5929 (7.9)	149/1649 (9.0)	29/521 (5.6)	69/1223 (5.6)	0.002
OSAS	254/5828 (4.4)	107/1608 (6.7)	36/497 (7.2)	47/1185 (4.0)	<0.001
Hyperthyroidism	266/5856 (4.5)	78/1605 (4.9)	21/514 (4.1)	59/1205 (4.9)	0.842
Hypothyroidism	511/5861 (8.7)	195/1606 (12.1)	50/516 (9.7)	117/1210 (9.7)	<0.001
Chronic kidney disease	413/5917 (7.0)	297/1637 (18.1)	111/518 (21.4)	322/1230 (26.2)	<0.001
CHA_2_DS_2_VASc, median (IQR)	2 (1–4)	4 (3–5)	4 (3–5)	4 (3–5)	<0.001
HAS‐BLED, median (IQR)	1 (1–2)	2 (1–2)	2 (1–3)	2 (1–3)	<0.001
Devices	428/5953 (7.2)	162/1662 (9.7)	57/523 (10.9)	268/1235 (21.7)	<0.001
CRT‐P/D	18/428 (2)	10/162 (6)	10/57 (17.6)	86/268 (32.0)	
ICD	72/428 (16.8)	17/162 (10.5)	11/57 (19.3)	113/268 (42.2)	
Optimal medical therapy	…	905/1662 (54.5)	313/523 (59.8)	807/1235 (65.3)	<0.001
ABC adherence	1386/4070 (34.1)	219/992 (22.1)	67/314 (21.3)	182/651 (28.0)	<0.001
A criterion adherence	3022/4070 (74.3)	768/992 (77.4)	260/314 (82.8)	520/651 (79.9)	<0.001
B criterion adherence	3380/4070 (83.0)	788/992 (79.4)	227/314 (72.3)	456/651 (70.0)	<0.001
C criterion adherence	2338/4070 (57.4)	353/992 (35.6)	116/314 (36.9)	287/651 (44.1)	<0.001

ABC indicates Atrial Fibrillation Better Care; AF, atrial fibrillation; CABG, coronary artery bypass grafting; COPD, chronic obstructive pulmonary disease; CRT‐P/D, cardiac resynchronization therapy–pacemaker/defibrillator; EHRA, European Heart Rhythm Association; ICD, implantable cardioverter‐defibrillator; IQR, interquartile range; LVEF, left ventricular ejection fraction; LVH, left ventricular hypertrophy; OSAS, obstructive sleep apnea syndrome; and PCI, percutaneous coronary intervention.

Regarding the management of these patients, a cardiac implantable electric device was more likely to be inserted in patients with HFrEF, while antiarrhythmic drugs were more likely prescribed in patients without HF.

Except for angiotensin receptor blockers and calcium channel blockers, which were more prescribed in patients with HFpEF, all the other concomitant medications were more commonly prescribed in patients with HFrEF (Table S1). OMT was increasingly used across the HF spectrum, being highest in HFrEF patients. Patients with HFpEF were more likely using only antiplatelet drugs than the other groups, and patients with HFpEF and patients with HFmrEF were more likely treated with vitamin K antagonists only, with the non–vitamin K antagonist OACs more commonly used in patients without HF (Table S1). Adherence to the ABC pathway was highest in patients with AF without HF, while patients with HFpEF and patients with HFmrEF were less likely to be adherent to the ABC pathway. Adherence to the “A” criterion was lower among patients without HF; conversely, adherence to both “B” and “C” criteria was highest in patients without HF.

### Major Adverse Outcomes According to EF Classes

**Figure 1:**
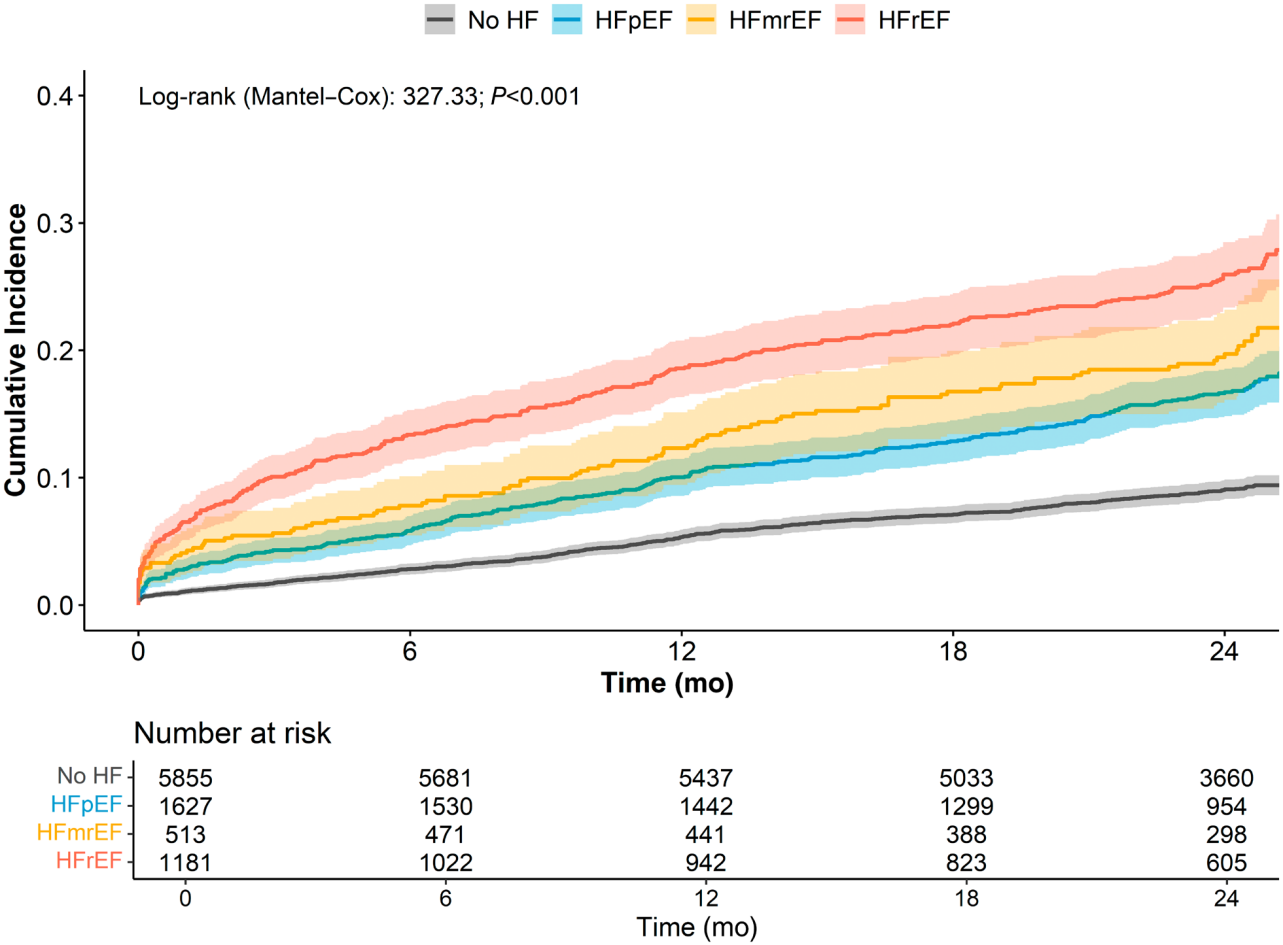
Kaplan–Meier curves for composite outcome according to HF categories. HF indicates heart failure; HFpEF, HF with preserved ejection fraction; HFmrEF, HF with mildly reduced ejection fraction; and HFrEF, HF with reduced ejection fraction.

During a median follow‐up time of 2.0 [IQR, 1.9–2.0] years, a total of 1434 (15.3%) events of the composite outcome occurred. Rates of the composite outcome, all‐cause death, and MACEs progressively increased from patients without HF to those with HFrEF (Table S3). Cumulative incidence of composite outcome was progressively higher from patients without HF to those with HFrEF (log‐rank, 327.33; *P*<0.001; Figure 1). In patients with HF, there were a total of 274 (8.0%) events of HF worsening/hospitalization. Rate of HF worsening/hospitalization was higher in patients with HFrEF (Table S4).

Multivariable Cox regression analysis (Table 2) showed that compared with patients without HF, all classes of HF were associated with increased risk of composite outcome occurrence, with highest figures observed for HFrEF (HR, 2.36 [95% CI, 2.00–2.78]). Similar results were found for all‐cause death and MACEs, with a higher magnitude. Continuous EF showed an inverse association with occurrence of adverse outcomes (Table 2). Regarding the occurrence of HF worsening/hospitalization, HFrEF was associated with a higher risk when compared with HFpEF, while no difference was found for HFmrEF (Table S4).

**Table 2:** Multivariable Cox Regression Analyses for Major Adverse Outcomes According to Ejection Fraction Classes

	Composite outcome		All‐cause death		MACEs	
	HR	95% CI	HR	95% CI	HR	95% CI
No HF	Ref.	Ref.	Ref.	Ref.	Ref.	Ref.
HFpEF	1.42	1.21–1.68	1.69	1.39–2.04	1.46	1.16–1.82
HFmrEF	1.78	1.41–2.25	2.15	1.65–2.81	1.59	1.14–2.22
HFrEF	2.36	2.00–2.78	2.97	2.46–3.59	2.66	2.15–3.30
Continuous LVEF (each 10%)	0.80	0.77–0.84	0.75	0.71–0.79	0.78	0.74–0.83

	Composite outcome		All‐cause death		MACEs	
	HR	95% CI	HR	95% CI	HR	95% CI
No HF	Ref.	Ref.	Ref.	Ref.	Ref.	Ref.
HF >40% LVEF	1.51	1.30–1.75	1.79	1.51–2.13	1.49	1.21–1.82
HF ≤40% LVEF	2.35	1.99–2.77	2.95	2.44–3.57	2.66	2.15–2.39

HF indicates heart failure; HFrEF, heart failure with reduced ejection fraction; HFmrEF, heart failure with mildly reduced ejection fraction; HFpEF, heart failure with preserved ejection fraction; HR, hazard ratio; LVEF, left ventricular ejection fraction; MACEs, major adverse cardiovascular events; and Ref., reference category.

In the simplified categorization, similar results were found, with higher rates of outcomes in patients with HF and reduced LVEF (Table S3) and an increasing cumulative incidence (Figure S1). For HF worsening/hospitalization, a higher rate was found for patients with HF and reduced LVEF (Table S4). Similarly, a progressively increasing association with risk of all outcomes was found for patients with HF with LVEF >40% and patients with LVEF ≤40% (Table 2), compared with those without HF. HF with LVEF ≤40% was also associated with HF worsening/hospitalization (Table S4).

Regression curve analysis found some evidence of a nonlinear relationship between LVEF and risk of composite outcome in the overall cohort (*P* for nonlinearity=0.052), with a risk that was exponentially higher for LVEF values <55%, while there was an inverse association with clinical events with LVEF above 55% (Figure S2A). The relationship also appeared nonlinear among patients with HF (*P*=0.040; Figure S2B).

### Impact of OMT on Major Adverse Outcomes

In patients with HF compliant to OMT, we found a lower rate of the composite outcome (21.9% versus 25.3%, *P*=0.022) and all‐cause death (14.7% versus 19.5%, *P*<0.001) compared with patients not compliant with OMT but not for MACEs (14.9% versus 16.3%, *P*=0.277). Cumulative incidence for the composite outcome was significantly higher for patients not compliant with OMT (Figure S3). Cox regression analysis showed that, after adjustments, OMT compliance was associated with a reduced risk of all the outcomes considered (Table 3). Compliance to OMT was also associated with a lower risk of HF worsening/hospitalization (Table S4).

**Table 3:** Impact of OMT and Adherence to ABC Pathway for Main Study Outcomes in Patients With HF

	Composite outcome		All‐cause death		MACEs	
	HR	95% CI	HR	95% CI	HR	95% CI
No OMT	Ref.	Ref.	Ref.	Ref.	Ref.	Ref.
OMT compliance	0.83	0.70–0.98	0.78	0.65–0.95	0.79	0.63–0.99

	Composite outcome		All‐cause death		MACEs	
	HR	95% CI	HR	95% CI	HR	95% CI
No HF	Ref.	Ref.	Ref.	Ref.	Ref.	Ref.
HF >40% LVEF OMT	1.45	1.21–1.75	1.65	1.33–2.05	1.48	1.16–1.90
HF >40% LVEF No OMT	1.59	1.32–1.92	1.97	1.59–2.44	1.53	1.18–1.99
HF ≤40% LVEF OMT	2.07	1.71–2.52	2.64	2.11–2.39	2.21	1.72–2.84
HF ≤40% LVEF No OMT	2.87	2.30–3.59	3.56	2.77–4.56	3.58	2.70–4.73

	Composite outcome		All‐cause death		MACEs	
	HR	95% CI	HR	95% CI	HR	95% CI
No ABC	Ref.	Ref.	Ref.	Ref.	Ref.	Ref.
ABC adherence	0.65	0.48–0.88	0.67	0.48–0.94	0.59	0.39–0.90

	Composite outcome		All‐cause death		MACEs	
	HR	95% CI	HR	95% CI	HR	95% CI
No HF	Ref.	Ref.	Ref.	Ref.	Ref.	Ref.
HF >40% LVEF ABC	1.04	0.70–1.55	1.19	0.76–1.87	1.19	0.70–2.00
HF >40% LVEF No ABC	1.47	1.22–1.78	1.78	1.43–2.21	1.46	1.13–1.90
HF ≤40% LVEF ABC	1.69	1.13–2.51	2.37	1.54–3.65	1.45	0.83–2.55
HF ≤40% LVEF No ABC	2.43	1.94–3.05	3.00	2.33–3.86	2.87	2.16–3.80

ABC indicates Atrial Fibrillation Better Care; HF, heart failure; HR, hazard ratio; LVEF, left ventricular ejection fraction; MACEs, major adverse cardiovascular events; OMT, optimal medical therapy; and Ref., referencecategory.

We then analyzed how compliance with OMT could mitigate the risk of outcomes according to the LVEF level. As shown in Figure S4, the cumulative risk for composite outcome varied depending on the combination of LVEF level and use of OMT, being the highest for patients HF with LVEF ≤40% not treated with OMT. Compared with those who were OMT noncompliant, both patients with HF with LVEF >40% and LVEF ≤40% who were treated with OMT showed a lower magnitude of association with risk of outcomes occurrence; HF patients with LVEF ≤40% not compliant with OMT showed the highest risk for all the outcomes (Table 3).

### Impact of ABC Pathway Adherence on Major Adverse Outcomes

In patients with HF, we found a lower rate of all the outcomes in those who were adherent to the ABC pathway, compared with those who were nonadherent (composite outcome, 17.3% versus 25.0%; *P*=0.001; all‐cause death, 11.3% versus 17.9%; *P*=0.001; MACEs, 11.5% versus 16.9%; *P*=0.006). Cumulative incidence for the composite outcome was significantly higher in patients who were not adherent to the ABC pathway (Figure S5). Cox regression analysis showed that management compliant with ABC was associated with a reduced risk of all the outcomes considered (Table 3). Adherence to ABC was not associated with difference in risk for HF worsening/hospitalization (Table S4).

We analyzed how adherence to the ABC pathway could mitigate the risk of outcomes according to LVEF levels. As shown in Figure S5, cumulative incidence for the composite outcome varied depending on the combination of LVEF level and adherence to the ABC pathway, being highest for patients with HFrEF who were not adherent to ABC. While patients with HFrEF treated adherent to ABC showed a lower magnitude of association with the risk of the 3 outcomes, adherence to the ABC pathway in those with HF and LVEF >40% exerted the greatest effect, with nonstatistically significant differences in risk of major outcomes compared with those without HF (Table 3). HF patients with LVEF ≤40% who were not adherent to the ABC pathway showed the higher risk for all the outcomes considered (Table 3).

**Figure 2:**
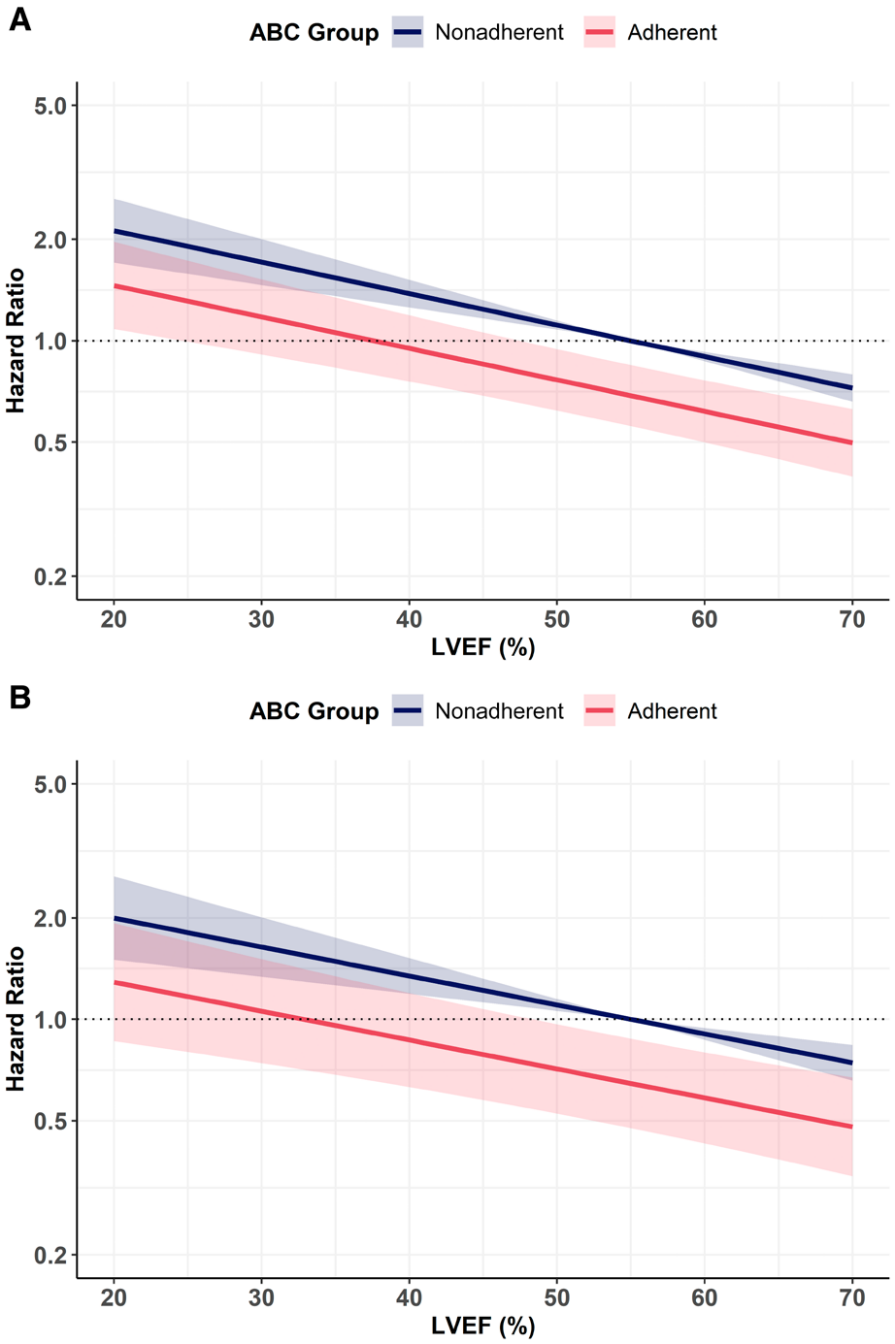
Regression curve analysis about LVEF and risk of composite outcome according to ABC adherence in the overall cohort and patients with HF. **A**, Overall cohort; **B**, Patients with HF. ABC indicatesAtrial Fibrillation Better Care; andLVEF, left ventricular ejection fraction.

Regression curve analysis showed that adherence to the ABC pathway was associated with reduction of the composite outcome across the spectrum of LVEF, both in the overall cohort (Figure 2A) and in patients with HF (Figure 2B).

### Sensitivity Analysis

**Figure 3:**
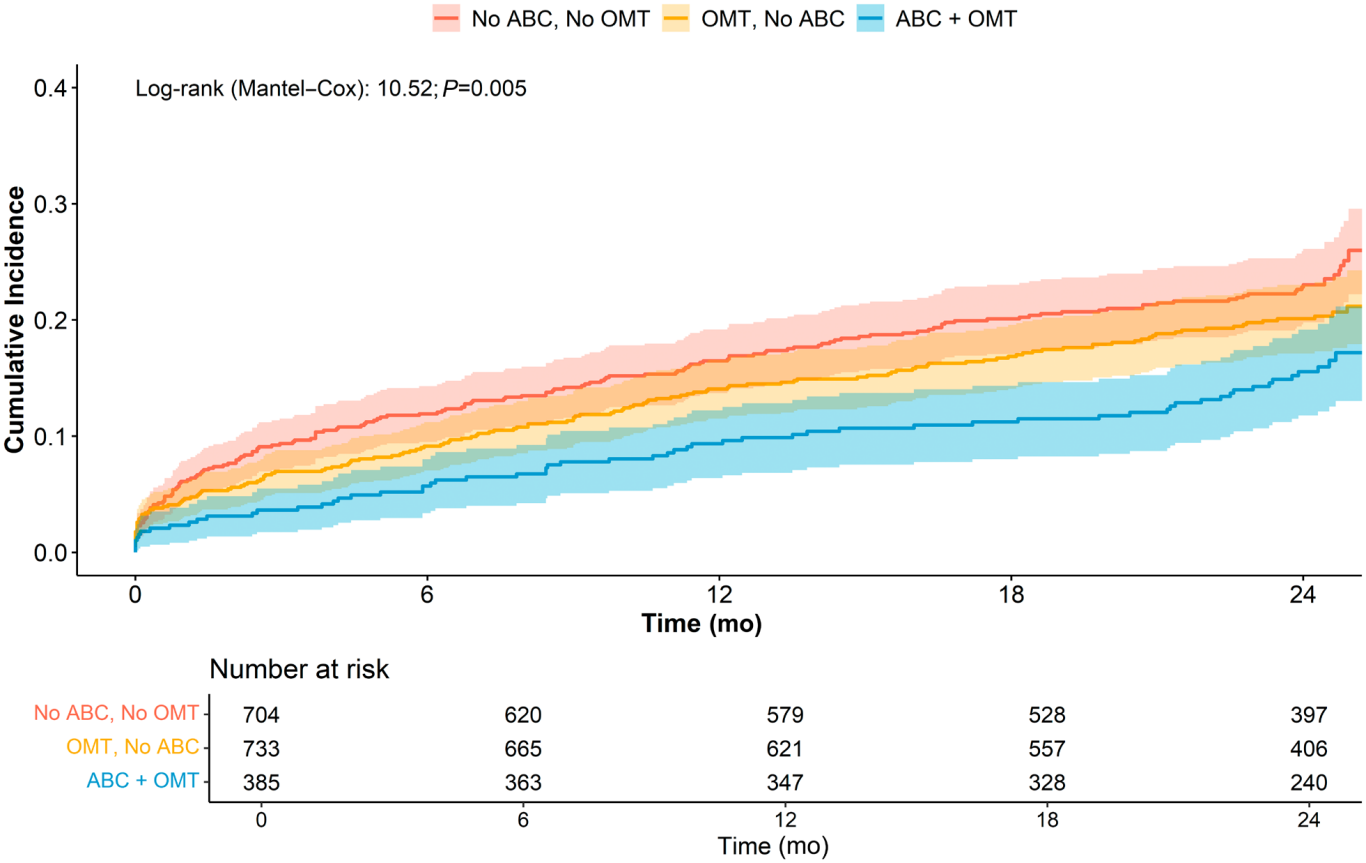
Kaplan–Meier curves for composite outcome according to OMT compliance and ABC adherence. ABC indicates Atrial Fibrillation Better Care; andOMT, optimal medical therapy.

We finally performed a sensitivity analysis to evaluate the effect of the interplay between compliance with OMT and adherence to the ABC pathway on the risk of composite outcome among patients with HF. Kaplan–Meier curve analysis (Figure 3) showed that treated patients adherent to both OMT and the ABC pathway had the lower cumulative incidence of the composite outcome. The additive value of OMT compliance and ABC pathway adherence was confirmed in the adjusted Cox regression analysis (OMT and ABC: HR, 0.67 [95% CI, 0.48–0.93]; OMT only: HR, 0.84 [95% CI, 0.66–1.09] versus no OMT and no ABC).

## DISCUSSION

In this analysis from a large European AF cohort, the principal findings were as follows: (1) in patients with AF, LVEF is inversely and nonlinearly associated with the risk of major outcomes values, both in patients with HF and in the overall cohort; (2) in patients with AF and HF, both OMT and ABC pathway adherence reduced the risk of major adverse events, with a magnitude of risk reduction greater for the ABC pathway; (3) compared with patients without HF, both OMT and ABC pathway mitigated the risk of major clinical outcomes irrespective of LVEF levels, and the effect of ABC pathway resulted in consistent risk reductions across the spectrum of LVEF in both the overall cohort of patients with AF and those with AF and HF.

Our analysis is the first to comprehensively assess the effect of an HF‐specific optimal management (as encompassed by guidelines‐defined OMT) and the ABC pathway on the risk of major outcomes in patients with AF and HF. We found that while OMT is effective in reducing the risk of the composite outcome of death and MACEs as well as the risk of both individual outcomes, the magnitude of the risk reduction is greater with a more comprehensive integrated care strategy, such as with the ABC pathway. Furthermore, we show how the effect of OMT in mitigating the risk of outcomes is mainly present in patients with HFrEF, while adherence to the ABC pathway exerts its effect irrespective of HF categorizations and, most importantly, consistently across the spectrum of LVEF, indicating that an integrated management is useful in the overall spectrum of HF disease.

The bidirectional association between AF and HF has been already extensively described; HF is found in 20% to 30% of patients with AF,^16^ and the coexistence of the 2 diseases exerts a synergistic detrimental effect on prognosis.^17^, ^18^ More recently, the relationship between HFpEF and AF has gained much attention, and several reports have underlined the close relationship between these 2 conditions.^16^, ^18^, ^19^ This has led to several studies investigating the impact of HF subtypes on AF, using LVEF cutoffs. On the other hand, few data are available on the risk of outcomes across the spectrum of LVEF in patients with AF and HF, and among the general AF population.

In our study, we have provided an extensive assessment of the association between HF classes, LVEF, and risk of major outcomes in patients with AF. We found that the relationship between LVEF and outcomes appears to be nonlinear, with risk increasing particularly when LVEF is <40%; this was also observed in the subgroup of patients with HF, emphasizing how prognosis is particularly poor when the LVEF becomes severely impaired. While several studies have shown heterogeneity regarding the impact of EF and EF classes on the risk of major adverse outcomes,^8^, ^20^, ^21^ as also underlined by experts,^22^ our findings appear to be supported by data coming from a large individual patient data meta‐analysis, which demonstrated how patients with HFrEF have a higher risk of all‐cause and cardiovascular death and that this risk is progressively higher with progressively lower EF.^23^

These observations may be explained by several pathophysiological hypotheses. HF with low LVEF leads to hemodynamic alterations due to the overload of volume and pressure, leading to macro‐ and microscopic modification of the myocardial conduction tissue^24^; on the other hand, the irregular rhythm imposed by AF worsens the overall cardiac function with complex mechanisms, including high heart rate and the absence of the hemodynamic contribution of the atrial systole.^25^ As proof of that, several trials enrolling patients with HF with reduced LVEF, such as the CASTLE‐AF (Catheter Ablation Versus Standard Conventional Therapy in Patients with Left Ventricular Dysfunction and Atrial Fibrillation) or CABANA (Catheter Ablation Versus Anti‐arrhythmic Drug Therapy for Atrial Fibrillation) trial, demonstrated the effectiveness of the rhythm control strategy.^26^, ^27^

The need for optimal management and a comprehensive approach has been repeatedly advocated for both HF and AF. Indeed, a previous analysis from the ESC‐EHRA EORP‐AF Pilot Registry has shown how patients with AF and HF experience poor outcomes despite high adoption of OAC,^28^ thus emphasizing the need for more comprehensive care in these patients.

Our findings have several clinical implications. First, as OMT is recommended by international guidelines for patients with HFrEF, our results confirm that patients with AF and HFpEF may require different strategies to improve their prognosis. Second, our findings on the effectiveness of the ABC pathway (and particularly regarding the effect across the spectrum of LVEF) suggest that these patients may benefit from a holistic approach, consistently with previous analyses.^29^ In our cohort, patients with AF and HFpEF presented with a significant burden of concurrent comorbidities, which may entail an overall high clinical‐complexity that requires a comprehensive approach for management.^30^, ^31^ Unsurprisingly, this evidence is consistent with other studies showing how AF and HFpEF are commonly found in the “aging” phenotype of HFpEF,^32^ also characterized by a significant burden of concurrent diseases.^33^

Furthermore, our data showed that an ABC pathway‐adherent management is also effective in reducing the risk of major outcomes in patients with AF with low LVEF. This result is particularly important, given the high risk of adverse events in these patients, as also confirmed in our cohort, with a nonlinear association between LVEF and the risk of the primary composite outcome. As OMT is part of the ABC pathway, which encompasses optimal management of concurrent comorbidities in AF, we showed how an integrated bundle of care leads to an even larger reduction in risk of major outcomes than the single, although optimal, management addressing a single component (ie, OMT for HF). Nonetheless, we show how only compliance to OMT was associated with a lower risk for HF worsening/hospitalization and not adherence to the ABC pathway per se. While this analysis is merely exploratory, we can hypothesize that because HF worsening/hospitalization is often related to fluid overload and acute ventricular dysfunction,^34^ the effect of OMT may be more directly related to those pathophysiological mechanisms responsible for acute decompensation. Nonetheless, the impact of adherence to the ABC pathway in reducing the risk of major adverse outcomes remains relevant, and larger in magnitude, than the mere compliance to OMT.

Taken together, our results suggest that the relationship between AF and HF is complex and perhaps difficult to capture by simple, albeit practical, categorization, and that the risk of major events follows a nonlinear, continuous increase as left ventricular function declines. As OMT is the cornerstone of treatment for patients with HF, a more comprehensive management approach according to the ABC pathway leads to a sharper improvement in the prognosis of all patients with AF and HF.

### Strengths and Limitations

In this analysis, we have provided a comprehensive analysis on the relationship between AF and HF, considering the continuous and nonlinear relationship between LVEF and AF, using a contemporary cohort of patients with AF. This allowed us to perform a more granular analysis on the interplay between AF and HF, beyond simple categorizations based on LVEF. Furthermore, this is the first analysis to specifically assess the impact of the ABC pathway in patients with AF and HF, showing its consistent effectiveness across the spectrum of LVEF.

Nevertheless, our study has several limitations. First, the observational design of the study may have influenced our ability to explore associations in this analysis. Also, the use of multiple comparisons imposes to use caution in interpreting and extending our results to the general patients with AF, particularly regarding the secondary exploratory outcomes. Considering the time of study enrollment, the proportion of patients receiving catheter ablation was low (511 [5.4%] patients at baseline), which could have influenced the risk of outcomes in the subset of patients with HF. Furthermore, our cohort was European based and may not reflect the overall AF population. Although we provided adjusted analyses, we cannot exclude the contribution of other unaccounted confounders, which may have influenced the results; therefore, our results should be interpreted with caution. In particular, we were not able to account for specific factors that could have influenced the physicians in prescribing/not prescribing a certain drug, as this was not investigated in the present study. The concept of the ABC pathway is not dependent on a “fixed” scheme of treatment but is rather adaptable to new evidence on the optimal management of comorbidities and cardiovascular optimization. Therefore, the use of the term *adherence* identifies the adherence to the diagnostic–therapeutic criteria of the ABC pathway, derived from baseline characteristics and overall clinical management data. Conversely, when referring to OMT “compliance,” its definition is based on the physicians' prescription of a combination of drugs, as reported from the cohort's baseline data, rather than the patients' compliance to prescribed drugs, which was not investigated in this study. Thus, it represents the most completed and tolerated HF therapies, according to clinical guidelines. As per guidelines, we are aware that OMT is mainly recommended for patients with HFrEF, although it is common in clinical practice to apply the same principles of drug therapy to those with HFpEF and HFmrEF. Indeed, OMT's effectiveness in reducing cardiovascular outcomes in these patients has been previously reported.^35^, ^36^ Moreover, a recent meta‐analysis showed that the combination of drugs used in our study to define OMT provided a strong trend for the reduction of all‐cause death and HF hospitalization.^37^ Because our study aimed to reflect real‐world clinical practice, we believe that our results depict the usual clinical approach, even though this remains a limitation and our conclusions about OMT use in HFpEF and HFmrEF must be taken with caution. Finally, several treatments have been introduced in clinical practice for HF management since our cohort was established, including angiotensin receptor–neprilysin inhibitor and sodium–glucose cotransporter 2 inhibitors, which were not part of clinical practice at the time of the present study. We believe that future studies, in newer cohorts in which both the new classes of drugs would be used, will provide additional evidence on the integration of HF OMT into the ABC pathway. Additionally, we have not considered the possible synergistic beneficial effect of cardiac implantable electronic devices with medical therapy,^38^ for which we did not have enough granularity of data.

## CONCLUSIONS

In a large contemporary European cohort of patients with AF, patients with HF showed higher risk of major adverse outcomes, with a continuous, nonlinear association between LVEF and risk of outcomes in both patients with HF and the overall cohort of patients with AF. OMT appears to be able to mitigate the risk in patients with HF and particularly with HFrEF, while a more comprehensive and integrated approach with the ABC pathway may lead to greater risk reduction across the whole spectrum of LVEF.

## Sources of Funding

Since the start of the EURObservational Research Programme, the following companies have supported the programme: Abbott Vascular Int. (2011–2021), Amgen Cardiovascular (2009–2018), AstraZeneca (2014–2021), Bayer (2009–2018), Boehringer Ingelheim (2009–2019), Boston Scientific Corporation (2009–2012), The Bristol Myers Squibb and Pfizer Alliance (2011–2016), The Alliance Daiichi Sankyo Europe GmbH and Eli Lilly and Company (2011–2017), Edwards (2016–2019), Gedeon Richter Plc. (2014–2017), Menarini Int. Op. (2009–2012), MSD‐Merck & Co. (2011–2014), Novartis Pharma AG (2014–2020), ResMed (2014–2016), Sanofi (2009–2011), SERVIER (2010–2021), and Vifor Pharma (2019–2022).

## Disclosures

Dr Romiti reports consultancy for Boehringer Ingelheim and an educational grant from Anthos, outside the submitted work. No fees are directly received personally. Dr Proietti is national leader of the AFFIRMO project on multimorbidity in atrial fibrillation, which has received funding from the European Union's Horizon 2020 research and innovation program under grant agreement number 899871. Dr Fauchier is a consultant or speaker for Bayer, BMS/Pfizer, Boehringer Ingelheim, Medtronic, Novartis, and XO. Dr Marin reports advisor fees from Boehringer Ingelheim; research grants from Ferrer; and speaker fees from Boehringer Ingelheim, Astra‐Zeneca, Pfizer, and Bayer. Dr Dan reports small speaker fees from Boehringer Ingelheim, Pfizer, Bayer, Sanofi, and Zentiva. Dr Potpara is a consultant for Bayer and Pfizer (no fees). Dr Boriani reports small speaker fees from Medtronic, Boston, Boehringer Ingelheim, and Bayer. Dr Lip has been consultant and speaker for BMS/Pfizer, Boehringer Ingelheim, Anthos, and Daiichi‐Sankyo. No fees are directly received personally. All the disclosures happened outside the submitted work. Dr Lip is a National Institute for Health and Care Research Senior Investigator and co‐principal investigator of the AFFIRMO project on multimorbidity in AF, which has received funding from the European Union's Horizon 2020 research and innovation program under grant agreement number 899871. All the disclosures occurred outside the submitted work. The remaining authors have no disclosures to report.

## Supporting information

Data S1
Tables S1–S4
Figures S1–S5
