# Supplementary material for: Optimal Medical Therapy for Heart Failure and Integrated Care in Patients With Atrial Fibrillation: A Report From the ESC‐EHRA EORP Atrial Fibrillation Long‐Term General Registry
Source: J Am Heart Assoc. 2024 Dec 20;14(1):e030499. doi: 10.1161/JAHA.123.030499 (PMC12054469; doi:10.1161/JAHA.123.030499)
Supplement: Supplementary file 1 — Data S1 Tables S1–S4 Figures S1–S5 [file JAH3-14-e030499-s001.zip › data supplement for publication.pdf]

Supplemental Material

EURObservational Research Programme Atrial Fibrillation (EORP-AF) Long-Term General Registry Committees and Investigators

Executive committee: G. Boriani (Chair), G.Y.H. Lip, L. Tavazzi, A. P. Maggioni, G-A. Dan, T. Potpara, M. Nabauer, F. Marin, Z. Kalarus, L. Fauchier, R. Ferrari, A. Shantsila.

Steering Committee (National Coordinators): A. Goda, *University Hospital Center "Mother Tereza", Tirana, Albania*; G. Mairesse, *Cliniques du Sud-Luxembourg, Arlon, Belgium;* T. Shalganov, *National Heart Hospital, Sofia, Bulgaria;* L. Antoniades, *Nicosia General Hospital, Latsia, Cyprus;* M. Taborsky, *University Hospital Olomouc, Olomouc, Czech Republic;* S. Riahi, *Aalborg University Hospital, Aalborg, Denmark;* P. Muda, *University of Tartu, Tartu, Estonia;* I. García Bolao, *Navarra Institute for Health Research, Pamplona, Spain;* O. Piot, *Centre Cardiologique du Nord, Saint-Denis, France;* M. Nabauer, *Ludwig-Maximilians-University, Munich, Germany;* K. Etsadashvili, *G. Chapidze Emergency Cardiology Center, Tbilisi, Georgia;* EN. Simantirakis, *University Hospital of Heraklion, School of Medicine, University of Crete, Heraklion, Crete, Greece;* M. Haim, *Soroka Medical Center, Beer Sheva, Israel;* A. Azhari, J. Najafian, *Cardiovascular Research Institute, Isfahan University of Medical Sciences, Isfahan, Iran;* M. Santini, *San Filippo Neri Hospital, Rome, Italy;* E. Mirrakhimov, *National Center of Cardiology and Internal Medicine, Bishkek, Kyrgyzstan;* K. Kulzida, *Scientific-Research Institute of Cardiology and Internal Diseases, Almaty, Republic of Kazakhstan;* A. Erglis, *Pauls Stradins Clinical University Hospital University of Latvia Riga Latvia;* L. Poposka, *University Clinic of Cardiology, Faculty of Medicine, Ss Cyril and Methodius University of Skopje, Skopje, Republic of Macedonia;* MR. Burg, *Mater Dei Hospital, Triq Dun Karm Psaila, Malta;* H. Crijns, Ö. Erküner, *Cardiovascular Research Institute Maastricht (CARIM), Maastricht University Medical Centre, Maastricht, The Netherlands;* D. Atar, *Oslo University Hospital Ullevål and Institute of Clinical Sciences, University of Oslo, Oslo, Norway;* R. Lenarczyk, *Silesian Center for Heart Disease, Zabrze, Poland;* M. Martins Oliveira, *Hospital Santa Marta, Lisbon, Portugal;* D. Shah, *Department of Medicine Specialities, University Hospital Geneva, Geneva, Switzerland;* G-A. Dan, *Colentina University Hospital, Bucharest, Romania;* E. Serdechnaya, *Northern State Medical University, Arkhangelsk, Russia;* T. Potpara, *Cardiology Clinic, Clinical Center of Serbia, Belgrade, Serbia;* E. Diker,

Başakşehir Çam and Sakura City Hospital, Istanbul, Turkey; G.Y.H. Lip, D. Lane; *City Hospital, University of Birmingham, Birmingham, United Kingdom.*

Investigators: **ALBANIA** Durrës: E. Zëra, Tirana: U. Ekmekçiu, V. Paparisto, M. Tase, Tirana: H. Gjergo, J. Dragoti, A. Goda, **BELGIUM** Bastogne: M. Ciutea, N. Ahadi, Z. el Husseini, M. Raepers, Gilly: J. Leroy, P. Haushan, A. Jourdan, Haine Saint Paul: C. Lepiece, Hasselt: L. Desteghe, J. Vijgen, P. Koopman, G. Van Genechten, H. Heidbuchel, Kortrijk: T. Boussy, M. De Coninck, H. Van Eeckhoutte, N. Bouckaert, La Louviere: A. Friart, J. Boreux, C. Arend, Liege: P. Evrard, Liège: L. Stefan, E. Hoffer, J. Herzet, M. Massoz, Liège: C. Celentano, M. Sprynger, L. Pierard, Liège: P. Melon, Overpelt: B. Van Hauwaert, C. Kuppens, D. Faes, D. Van Lier, A. Van Dorpe, Waremme: A. Gerardy, Yvoir: O. Deceuninck, O. Xhaet, F. Dormal, E. Ballant, D. Blommaert, **BULGARIA** Pleven: D. Yakova, M. Hristov, T. Yncheva, N. Stancheva, S. Tisheva, Plovdiv: M. Tokmakova, F. Nikolov, D. Gencheva, Sofia: T. Shalganov, B. Kunev, M. Stoyanov, Sofia: D. Marchov, V. Gelev, V. Traykov, Varna: A. Kisheva, H. Tsvyatkov, R. Shtereva, S. Bakalska-Georgieva, S. Slavcheva, Y. Yotov, **CZECH REPUBLIC** Ústí nad Labem: M. Kubíčková, **DENMARK** Aalborg: A. Marni Joensen, A. Gammelmark, L. Hvilsted Rasmussen, P. Dinesen, S. Riahi, S. Krogh Venø, B. Sorensen, A. Korsgaard, K. Andersen, C. Fragtrup Hellum, Esbjerg: A. Svenningsen, O. Nyvad, P. Wiggers, Herning: O. May, A. Aarup, B. Graversen, L. Jensen, M. Andersen, M. Svejgaard, S. Vester, S. Hansen, V. Lynggaard, Madrid: M. Ciudad, Tallinn: R. Vettus, Tartu: P. Muda, **ESTONIA** Elche, Alicante: A. Maestre, Toledo: S. Castaño, **FRANCE** Abbeville: S. Cheggour, Abbeville: J. Poulard, V. Mouquet, S. Leparrée, Aix-en-Provence: J. Bouet, J. Taieb, Amiens: A. Doucy, H. Duquenne, Angers: A. Furber, J. Dupuis, J. Rautureau, Aurillac: M. Font, P. Damiano, Avignon Cedex: M. Lacrimini, Brest: J. Abalea, S. Boismal, T. Menez, J. Mansourati, Chartres: G. Range, H. Gorka, C. Laure, C. Vassalière, Creteil: N. Elbaz, N. Lellouche, K. Djouadi, Montpellier: F. Roubille, D. Dietz, J. Davy, Nimes: M. Granier, P. Winum, C. Leperchois-Jacquey, Paris: H. Kassim, E. Marijon, J. Le Heuzey, Paris: J. Fedida, C. Maupain, C. Himbert, E. Gandjbakhch, F. Hidden-Lucet, G. Duthoit, N. Badenco, T. Chastre, X. Waintraub, M. Oudihat, J. Lacoste, C. Stephan, Pau: H. Bader, N. Delarche, L. Giry, Pessac: D. Arnaud, C. Lopez, F. Boury, I. Brunello, M. Lefèvre, R. Mingam, M. Haissaguerre, Rennes: M. Le Bidan, D. Pavin, V. Le Moal, C. Leclercq, Saint Denis: O. Piot, T. Beitar, Saint Etienne: I. Martel, A. Schmid, N. Sadki, C. Romeyer-Bouchard, A. Da Costa, Tours: I. Arnault, M. Boyer, C. Piat, L. Fauchier, **FYR MACEDONIA** Bitola: N. Lozance, S. Nastevska, Ohrid: A. Doneva, B. Fortomaroska Milevska, B. Sheshoski, K. Petroska, N. Taneska, N. Bakrecheski, Skopje: K. Lazarovska, S. Jovevska, V. Ristovski, A. Antovski, Skopje: E. Lazarova, I. Kotlar, J. Taleski, L. Poposka, S. Kedev, Skopje: N. Zlatanovik, Štip: S. Jordanova, T. Bajraktarova Proseva, S. Doncovska, **GEORGIA** Tbilisi: D. Maisuradze, A. Esakia, E. Sagirashvili, K.

Lartsuliani, N. Natelashvili, N. Gumberidze, R. Gvenetadze, Tbilisi: K. Etsadashvili, N. Gotonelia, N. Kuridze, Tbilisi: G. Papiashvili, I. Menabde, **GERMANY** Aachen: S. Glöggler, A. Napp, C. Lebherz, H. Romero, K. Schmitz, M. Berger, M. Zink, S. Köster, J. Sachse, E. Vonderhagen, G. Soiron, K. Mischke, Bad Reichenhall: R. Reith, M. Schneider, Berlin: W. Rieker, Biberach: D. Boscher, A. Taschareck, A. Beer, Boppard: D. Oster, Brandenburg: O. Ritter, J. Adamczewski, S. Walter, Chemnitz: A. Frommhold, E. Luckner, J. Richter, M. Schellner, S. Landgraf, S. Bartholome, Chemnitz: R. Naumann, J. Schoeler, Dachau: D. Westermeier, F. William, K. Wilhelm, M. Maerkl, Detmold: R. Oekinghaus, M. Denart, M. Kriete, U. Tebbe, Ebersbach: T. Scheibner, Erlangen: M. Gruber, A. Gerlach, C. Beckendorf, L. Anneken, M. Arnold, S. Lengerer, Z. Bal, C. Uecker, H. Förtsch, S. Fechner, V. Mages, Friedberg: E. Martens, H. Methe, Göttingen: T. Schmidt, Hamburg: B. Schaeffer, B. Hoffmann, J. Moser, K. Heitmann, S. Willems, S. Willems, Hartmannsdorf: C. Klaus, I. Lange, Heidelberg: M. Durak, E. Esen, Itzehoe: F. Mibach, H. Mibach, Kassel: A. Utech, Kirchzarten: M. Gabelmann, R. Stumm, V. Ländle, Koblenz: C. Gartner, C. Goerg, N. Kaul, S. Messer, D. Burkhardt, C. Sander, R. Orthen, S. Kaes, Köln: A. Baumer, F. Dodos, Königsbrück: A. Barth, G. Schaeffer, Leisnig: J. Gaertner, J. Winkler, Leverkusen: A. Fahrig, J. Aring, I. Wenzel, Limburg: S. Steiner, A. Kliesch, E. Kratz, K. Winter, P. Schneider, Ludwigsburg: A. Haag, I. Mutscher, R. Bosch, Markkleeberg: J. Taggeselle, S. Meixner, Meissen: A. Schnabel, Meppen: A. Shamalla, H. Hötz, A. Korinth, Merzig: C. Rheinert, Moosburg: G. Mehltretter, Mühldorf: B. Schön, N. Schön, A. Starflinger, E. Englmann, Munich: G. Baytok, T. Laschinger, G. Ritscher, Munich: A. Gerth, Münster: D. Dechering, L. Eckardt, Nienburg: M. Kuhlmann, N. Proskynitopoulos, Paderborn: J. Brunn, K. Foth, Pirna: C. Axthelm, H. Hohensee, K. Eberhard, S. Turbanisch, Plauen: N. Hassler, A. Koestler, Riesa: G. Stenzel, Riesa: D. Kschiwan, M. Schwefer, S. Neiner, S. Hettwer, Rotenburg a.d. Fulda: M. Haeussler-Schuchardt, R. Degenhardt, S. Sennhenn, S. Steiner, Starnberg: M. Brendel, Westerstede: A. Stoehr, W. Widjaja, S. Loehndorf, A. Logemann, J. Hoskamp, J. Grundt, Zorneding: M. Block, Zwiesel: R. Ulrych, A. Reithmeier, V. Panagopoulos, **ITALY** Bologna: C. Martignani, D. Bernucci, E. Fantecchi, I. Diemberger, M. Ziacchi, M. Biffi, P. Cimaglia, J. Frisoni, G. Boriani, Firenze: I. Giannini, S. Boni, S. Fumagalli, S. Pupo, A. Di Chiara, P. Mirone, Modena: E. Fantecchi, G. Boriani, F. Pesce, C. Zoccali, V.L. Malavasi, **KAZAKHSTAN** Almaty: A. Mussagaliyeva, B. Ahyt, Z. Salihova, K. Koshum-Bayeva, **KYRGYZSTAN** Bishkek: A. Kerimkulova, A. Bairamukova, E. Mirrakhimov, **LATVIA** Riga: B. Lurina, R. Zuzans, S. Jegere, I. Mintale, K. Kupics, K. Jubele, A. Erglis, O. Kalejs, **MALTA** Birkirkara: K. Vanhear, M. Burg, M. Cachia, E. Abela, S. Warwicker, T. Tabone, R. Xuereb, **MONTENEGRO** Podgorica: D. Asanovic, D. Drakalovic, M. Vukmirovic, N. Pavlovic, L. Music, N. Bulatovic, A. Boskovic, **NETHERLANDS** Almere: H. Uiterwaal, N. Bijsterveld, Amsterdam: J. De Groot, J. Neefs, N. van den Berg, F. Piersma, A. Wilde, Delfzijl: V. Hagens, Enschede: J. Van Es, J. Van

Opstal, B. Van Rennes, H. Verheij, W. Breukers, Heerenveen: G. Tjeerdsma, R. Nijmeijer, D. Wegink, R. Binnema, Hengelo: S. Said, Maastricht: Ö. Erküner, S. Philippens, W. van Doorn, H. Crijns, Rotterdam: T. Szili-Torok, R. Bhagwandien, P. Janse, A. Muskens, s-Hertogenbosch: M. van Eck, R. Gevers, N. van der Ven, Venlo: A. Duygun, B. Rahel, J. Meeder, **NORWAY** Oslo: A. Vold, C. Holst Hansen, I. Engset, D. Atar, **POLAND** Bytom: B. Dyduch-Fejklowicz, E. Koba, M. Cichocka, Cieszyn: A. Sokal, A. Kubicius, E. Pruchniewicz, Gliwice: A. Kowalik-Sztylc, W. Czapla, Katowice: I. Mróz, M. Kozlowski, T. Pawlowski, M. Tendera, Katowice: A. Winiarska-Filipek, A. Fidyk, A. Slowikowski, M. Haberka, M. Lachor-Broda, M. Biedron, Z. Gasior, Kielce: M. Kołodziej, M. Janion, Kielce: I. Gorczyca-Michta, B. Wozakowska-Kaplon, Łódź: M. Stasiak, P. Jakubowski, T. Ciurus, J. Drozdz, Łódź: M. Simiera, P. Zajac, T. Wcislo, P. Zycinski, J. Kasprzak, Nysa: A. Olejnik, E. Harc-Dyl, J. Miarka, M. Pasieka, M. Ziemińska-Łuć, W. Bujak, Opoczno: A. Śliwiński, A. Grech, J. Morka, K. Petrykowska, M. Prasał, Opole: G. Hordyński, P. Feusette, P. Lipski, A. Wester, Radlin: W. Streb, Rzeszów: J. Romanek, P. Woźniak, M. Chlebuś, P. Szafarz, W. Stanik, Szczecin: M. Zakrzewski, J. Kaźmierczak, Szczecin: A. Przybylska, E. Skorek, H. Błaszczyk, M. Stępień, S. Szabowski, W. Krysiak, M. Szymańska, Tarnów: J. Karasiński, J. Blicharz, M. Skura, Warsaw: K. Hałas, L. Michalczyk, Z. Orski, K. Krzyżanowski, A. Skrobowski, Warsaw: L. Zieliński, M. Tomaszewska-Kiecana, M. Dłużniewski, Warsaw: M. Kiliszek, M. Peller, M. Budnik, P. Balsam, G. Opolski, A. Tymińska, K. Ozierański, A. Wancerz, Warsaw: A. Borowiec, E. Majos, R. Dabrowski, H. Szwed, Zabrze: A. Musialik-Lydka, Zabrze: A. Leopold-Jadczyk, E. Jedrzejczyk-Patej, M. Koziel, R. Lenarczyk, M. Mazurek, Z. Kalarus, Zabrze: K. Krzemien-Wolska, P. Starosta, E. Nowalany-Kozielska, Zakopane: A. Orzechowska, M. Szpot, M. Staszel, **PORTUGAL** Almada: S. Almeida, H. Pereira, L. Brandão Alves, R. Miranda, L. Ribeiro, Carnaxide Lisboa: F. Costa, F. Morgado, P. Carmo, P. Galvao Santos, R. Bernardo, P. Adragão, Santarém: G. Ferreira da Silva, M. Peres, M. Alves, M. Leal, Vila Real: A. Cordeiro, P. Magalhães, P. Fontes, S. Leão, Viseu: A. Delgado, A. Costa, B. Marmelo, B. Rodrigues, D. Moreira, J. Santos, L. Santos, **ROMANIA** Arad: A. Terchet, D. Darabantiu, S. Mercea, V. Turcin Halka, A. Pop Moldovan, Brasov: A. Gabor, B. Doka, G. Catanescu, H. Rus, L. Oboroceanu, E. Bobescu, Bucharest: R. Popescu, A. Dan, A. Buzea, I. Daha, G. Dan, I. Neuhoff, Bucharest: M. Baluta, R. Ploesteanu, N. Dumitrache, M. Vintila, Bucharest: A. Daraban, C. Japie, E. Badila, H. Tewelde, M. Hostiuc, S. Frunza, E. Tintea, D. Bartos, Bucharest: A. Ciobanu, I. Popescu, N. Toma, C. Gherghinescu, D. Cretu, N. Patrascu, C. Stoicescu, C. Udroiu, G. Bicescu, V. Vintila, D. Vinereanu, M. Cinteza, R. Rimbas, Iași: M. Grecu, Oradea: A. Cozma, F. Boros, M. Ille, O. Tica, R. Tor, A. Corina, A. Jeewooth, B. Maria, C. Georgiana, C. Natalia, D. Alin, D. Dinu-Andrei, M. Livia, R. Daniela, R. Larisa, S. Umaar, T. Tamara, M. Ioachim Popescu, Târgu Mureș: D. Nistor, I. Sus, O. Coborosanu, Timișoara: N. Alina-Ramona, R. Dan, L. Petrescu, Timișoara: G. Ionescu, I. Popescu, C.

Vacarescu, E. Goanta, M. Mangea, A. Ionac, C. Mornos, D. Cozma, S. Pescariu, **RUSSIAN FEDERATION** Arkhangelsk: E. Solodovnicova, I. Soldatova, J. Shutova, L. Tjuleneva, T. Zubova, V. Uskov, Arkhangelsk: D. Obukhov, G. Rusanova, Arkhangelsk: I. Soldatova, N. Isakova, S. Odinsova, T. Arhipova, Arkhangelsk: E. Kazakevich, E. Serdechnaya, O. Zavyalova, Saint-Petersburg: T. Novikova, Saint-Petersburg: I. Riabaia, S. Zhigalov, Saint-Petersburg: E. Drozdova, I. Luchkina, Y. Monogarova, Vladivostok: D. Hegya, L. Rodionova, L. Rodionova, V. Nevzorova, Vladivostok: I. Soldatova, O. Lusanova, **SERBIA** Belgrade: A. Arandjelovic, D. Toncev, M. Milanov, N. Sekularac, Belgrade: M. Zdravkovic, S. Hinic, S. Dimkovic, T. Acimovic, J. Saric, Belgrade: M. Polovina, T. Potpara, B. Vujisic-Tesic, M. Nedeljkovic, Belgrade: M. Zlatar, M. Asanin, Belgrade: V. Vasic, Z. Popovic, Belgrade: D. Djikic, M. Sipic, V. Peric, B. Dejanovic, N. Milosevic, Belgrade: A. Stevanovic, A. Andric, B. Pencic, M. Pavlovic-Kleut, V. Celic, Kragujevac: M. Pavlovic, M. Petrovic, M. Vuleta, N. Petrovic, S. Simovic, Z. Savovic, S. Milanov, G. Davidovic, V. Iric-Cupic, Niška Banja: D. Simonovic, M. Stojanovic, S. Stojanovic, V. Mitic, V. Ilic, D. Petrovic, M. Deljanin Ilic, S. Ilic, V. Stoickov, Pirot: S. Markovic, Šabac:S. Kovacevic. **SPAIN** Alicante: A. García Fernandez, Benalmadena: A. Perez Cabeza, Córdoba: M. Anguita, Granada: L. Tercedor Sanchez, Huarte: E. Mau, J. Loayssa, M. Ayarra, M. Carpintero, Madrid: I. Roldán Rabadan, Murcia: M. Leal, Murcia: M. Gil Ortega, Murcia: A. Tello Montoliu, E. Orenes Piñero, S. Manzano Fernández, F. Marín, A. Romero Aniorte, A. Veliz Martínez, M. Quintana Giner, Pamplona: G. Ballesteros, M. Palacio, O. Alcalde, I. García-Bolao, San Juan de Alicante: V. Bertomeu Gonzalez, Santiago de Compostela: F. Otero-Raviña, J. García Seara, J. Gonzalez Juanatey, **SWITZERLAND** Geneva: N. Dayal, P. Maziarski, P. Gentil-Baron, D. Shah, **TURKEY** Adana: M. Koç, Afyon: E. Onrat, I. E. Dural, Ankara: K. Yilmaz, B. Özin, Ankara: S. Tan Kurklu, Y. Atmaca, Ankara: U. Canpolat, L. Tokgozoglu, Ankara: A. K. Dolu, B. Demirtas, D. Sahin, Ankara: O. Ozcan Celebi, E. Diker, Antalya: G. Gagirci, Bayraklı/Izmir: U.O.Turk, Bursa: H. Ari, Diyarbakır: N. Polat, N. Toprak, Gaziantep: M. Sucu, Görükle-Bursa: O. Akin Serdar, Istanbul: A. Taha Alper, Istanbul: A. Kepez, Istanbul: Y. Yuksel, Kurupelit - Samsun: A. Uzunselvi, S. Yuksel, M. Sahin, Merkez/Düzce: O. Kayapinar, Mersin: T. Ozcan, Sivas: H. Kaya, M. B. Yilmaz, Trabzon: M. Kutlu, Yüreğir-Adana: M. Demir, **UNITED KINGDOM** Barnstaple: C. Gibbs, S. Kaminskiene, M. Bryce, A. Skinner, G. Belcher, J. Hunt, L. Stancombe, B. Holbrook, C. Peters, S. Tettersell, Birmingham: A. Shantsila, D. Lane, K. Senoo, M. Proietti, K. Russell, P. Domingos, S. Hussain, J. Partridge, R. Haynes, S. Bahadur, R. Brown, S. McMahon, G. Y H Lip, Blackburn: J. McDonald, K. Balachandran, R. Singh, S. Garg, H. Desai, K. Davies, W. Goddard, Blackpool: G. Galasko, I. Rahman, Y. Chua, O. Payne, S. Preston, O. Brennan, L. Pedley, C. Whiteside, C. Dickinson, J. Brown, K. Jones, L. Benham, R. Brady, Carlisle: L. Buchanan, A. Ashton, H. Crowther, H. Fairlamb, S. Thornthwaite, C. Relph, A. McSkeane, U. Poultney, N. Kelsall, P. Rice, T. Wilson, Chertsey: M. Wrigley, R.

Kaba, T. Patel, E. Young, J. Law, Cramlington: C. Runnett, H. Thomas, H. McKie, J. Fuller, S. Pick, Exeter: A. Sharp, A. Hunt, K. Thorpe, C. Hardman, E. Cusack, L. Adams, M. Hough, S. Keenan, A. Bowring, J. Watts, Great Yarmouth: J. Zaman, K. Goffin, H. Nutt, Harrogate: Y. Beerachee, J. Featherstone, C. Mills, J. Pearson, L. Stephenson, Huddersfield: S. Grant, A. Wilson, C. Hawksworth, I. Alam, M. Robinson, S. Ryan, Macclesfield: R. Egdell, E. Gibson, M. Holland, D. Leonard, Maidstone: B. Mishra, S. Ahmad, H. Randall, J. Hill, L. Reid, M. George, S. McKinley, L. Brockway, W. Milligan, Manchester: J. Sobolewska, J. Muir, L. Tuckis, L. Winstanley, P. Jacob, S. Kaye, L. Morby, Nottingham: A. Jan, T. Sewell, Poole: C. Boos, B. Wadams, C. Cope, P. Jefferey, Portsmouth: N. Andrews, A. Getty, A. Suttling, C. Turner, K. Hudson, R. Austin, S. Howe, Redhill: R. Iqbal, N. Gandhi, K. Brophy, P. Mirza, E. Willard, S. Collins, N. Ndlovu, Rhyl: E. Subkovas, V. Karthikeyan, L. Waggett, A. Wood, A. Bolger, J. Stockport, L. Evans, E. Harman, J. Starling, L. Williams, V. Saul, Salisbury: M. Sinha, L. Bell, S. Tudgay, S. Kemp, J. Brown, L. Frost, Shrewsbury: T. Ingram, A. Loughlin, C. Adams, M. Adams, F. Hurford, C. Owen, C. Miller, D. Donaldson, H. Tivenan, H. Button, South Shields: A. Nasser, O. Jhagra, B. Stidolph, C. Brown, C. Livingstone, M. Duffy, P. Madgwick, Southampton: P. Roberts, E. Greenwood, L. Fletcher, M. Beveridge, S. Earles, Taunton: D. McKenzie, D. Beacock, M. Dayer, M. Seddon, D. Greenwell, F. Luxton, F. Venn, H. Mills, J. Rewbury, K. James, K. Roberts, L. Tonks, Torquay: D. Felmeden, W. Taggu, A. Summerhayes, D. Hughes, J. Sutton, L. Felmeden, Watford: M. Khan, E. Walker, L. Norris, L. O'Donohoe, Weston-super-Mare: A. Mozid, H. Dymond, H. Lloyd-Jones, G. Saunders, D. Simmons, D. Coles, D. Cotterill, S. Beech, S. Kidd, Wolverhampton: B. Wrigley, S. Petkar, A. Smallwood, R. Jones, E. Radford, S. Milgate, S. Metherell, V. Cottam, Yeovil: C. Buckley, A. Broadley, D. Wood, J. Allison, K. Rennie, L. Balian, L. Howard, L. Pippard, S. Board, T. Pitt-Kerby.

Table S1: Pharmacological Therapy according to Presence and Type of Heart Failure

N= 9373	No HF n= 5953	HFpEF n= 1662	HFmrEF n= 523	HFrEF n= 1235	p
**Any antiarrhythmic drug**, n (%)	1752/5927 (29.6)	368/1653 (22.3)	115/523 (22.0)	290/1230 (23.6)	<0.001
**ACE-inhibitors**, n (%)	2135/5929 (36.0)	738/1653 (44.6)	261/522 (50.0)	713/1233 (57.8)	<0.001
**ARBs**, n (%)	1129/5926 (19.1)	398/1653 (24.1)	113/523 (21.6)	181/1233 (14.7)	<0.001
**Beta-blockers**, n (%)	3855/5928 (65.0)	1210/1654 (73.2)	402/523 (76.9)	970/1232 (78.7)	<0.001
**MRAs**, n (%)	400/5926 (6.7)	414/1653 (25.0)	196/522 (37.5)	610/1232 (49.5)	<0.001
**Diuretics**, n (%)	2140/5925 (36.1)	1099/1653 (66.5)	413/522 (79.1)	1032/1233 (83.7)	<0.001
**Digoxin**, n (%)	522/5926 (8.8)	312/1653 (18.9)	122/523 (23.3)	391/1232 (31.7)	<0.001
**Calcium channel blockers**, n (%)	1068/5925 (18.0)	299/1655 (18.1)	89/523 (17.0)	111/1233 (9.0)	<0.001
**Non-DHP CCB**, n (%)	334/5927 (5.6)	94/1655 (5.7)	25/523 (4.8)	50/1233 (4.1)	0.129
**Statins**, n (%)	2309/5927 (39.0)	779/1653 (47.1)	245/523 (46.8)	610/1232 (49.5)	<0.001
**Oral antidiabetics**, n (%)	765/5929 (12.9)	303/1656 (18.3)	115/523 (22.0)	249/1233 (20.2)	<0.001
**Insulin**, n (%)	215/5926 (3.6)	126/1655 (7.6)	52/523 (9.9)	116/1233 (9.4)	<0.001
**Antithrombotic treatment**, n (%)					<0.001
None	502/5948 (8.4)	81/1659 (4.9)	19/523 (3.6)	56/1235 (4.5)	
Only Antiplatelets	343/5948 (5.8)	152/1659 (9.2)	37/523 (7.1)	93/1235 (7.5)	
Only VKAs	2273/5948 (38.2)	791/1659 (47.7)	247/523 (47.2)	543/1235 (44.0)	
Only NOACs	2250/5948 (37.8)	431/1659 (26.0)	157/523 (30.0)	276/1235 (22.3)	
Any OAC + Antiplatelets	580/5948 (9.8)	204/1659 (12.3)	63/523 (12.0)	267/1235 (21.6)	

Legend: ACE= angiotensin converting enzyme; ARB= angiotensin receptor blocker; CCB= calcium channel blockers; DHP= Dihydropyridine; MRAs= Mineralcorticoid Receptor Antagonists; NOAC= Non-Vitamin K Oral Anticoagulants; OAC= oral anticoagulants; VKA= Vitamin K antagonist.

Table S2: Baseline Characteristics of the Study Population according to LVEF Simplified Classification

	No HF<br>n= 5953	HF >40% LVEF<br>n= 2185	HF ≤40% LVEF<br>n= 1235	p
**Age (years)**, median (IQR)	69 (61-76)	74 (66-79)	71 (63-78)	<0.001
**Female**, n (%)	2307/5953 (38.8)	1075/2185 (49.2)	358/1235 (29)	<0.001
**BMI (kg/m^2)**, median (IQR)	27.70 [24.90, 31.10]	27.70 [24.80, 31.60]	27.10 [24.20, 30.55]	<0.001
**Region of enrolment**[§], n (%)				<0.001
Western Europe	2225/5953 (37.4)	720/2185 (33.0)	268/1235 (21.7)	
Southern Europe	2153/5953 (36.2)	684/2185 (31.3)	436/1235 (35.3)	
Northern Europe	1020/5953 (17.1)	104/2185 (4.8)	136/1235 (11.0)	
Eastern Europe	555/5953 (9.3)	677/2185 (31.0)	395/1235 (32.0)	
**AF type**, n (%)	5826/5953 (97.8)		1220/1225 (98.8)	<0.001
First diagnosed	1027/5826 (17.6)	258/2170 (11.9)	178/1220 (14.6)	
Paroxysmal	1763/5826 (30.3)	466/2170 (21.5)	156/1220 (12.8)	
Persistent	1278/5826 (21.9)	349/2170 (16.1)	208/1220 (17.0)	
Long-standing persistent	242/5826 (4.2)	127/2170 (5.9)	55/1220 (4.5)	
Permanent	1516/5826 (26.0)	970/2170 (44.7)	623/1220 (51.1)	
**Hypertension**, n (%)	3486/5916 (58.9)	1516/2172 (69.8)	729/1224 (59.6)	<0.001
**Diabetes mellitus**, n (%)	1116/5919 (18.9)	646/2178 (29.7)	376/1224 (30.7)	<0.001
**Lipid disorder**, n (%)	2201/5723 (38.5)	1002/2085 (48.1)	533/1186 (44.9)	<0.001
**Smoking (current)**, n (%)	535/5477 (9.8)	141/2083 (6.8)	136/1138 (12.0)	<0.001
**Alcohol (any intake),** n (%)	2046/5173 (39.6)	570/2022 (28.2)	383/1091 (35.1)	<0.001
**NYHA class n, (%)**	-			<0.001
I	-	373/2184 (17.1)	110/1234 (8.9)	
II	-	1162/2184 (53.2)	500/1234 (40.5)	

III	-	564/2184 (25.8)	501/1234 (40.6)	
IV	-	85/2184 (3.9)	123/1234 (10.0)	
**EHRA Score (III-IV) 3-4, n (%)**	974/5953 (16.4)	469/2185 (21.5)	357/1235 (28.9)	<0.001
**Coronary artery disease**, n (%)	1222/5810 (21.0)	742/1976 (37.6)	562/1128 (49.8)	<0.001
Previous MI	492/1222 (40.3)	275/742 (37.1)	347/562 (61.7)	<0.001
Previous angina	343/1222 (28.1)	289/742 (38.9)	159/562 (28.3)	<0.001
Previous PCI	574/1222 (47.0)	260/742 (35.0)	234/562 (41.6)	<0.001
Previous CABG	245/1222 (20.0)	126/742 (17.0)	112/562 (19.9)	0.211
**LVEF (%), median (IQR)**	60 (53-64)	55 (50-61)	31 (25-38)	<0.001
**LVH, n (%)**	1173/4816 (24.4)	795/2038 (39.0)	280/1141 (24.5)	<0.001
**Valvular alterations, n (%)**	2404/5848 (41.1)	1413/2166 (65.2)	893/1226 (72.8)	<0.001
**Any CMP, n (%)**	380/5953 (6.4)	280/2185 (12.8)	686/1235 (55.5)	<0.001
**Previous TE events**, n (%)	636/5927 (10.7)	277/2159 (12.8)	167/1210 (13.8)	0.004
**Haemorrhagic events**, n (%)	241/5922 (4.1)	167/2162 (7.7)	80/1213 (6.6)	<0.001
**Peripheral vascular disease**, n (%)	325/5887 (5.5)	259/2129 (12.2)	153/1188 (12.9)	<0.001
**Liver disease**, n (%)	92/5934 (1.6)	83/2166 (3.8)	77/1229 (6.3)	<0.001
**COPD**, n (%)	381/5913 (6.4)	287/2166 (13.3)	151/1225 (12.3)	<0.001
**Dementia**, n (%)	37/5943 (0.6)	42/2176 (1.9)	38/1224 (3.1)	<0.001
**Anaemia**, n (%)	176/5942 (3.0)	187/2176 (8.6)	133/1232 (10.8)	<0.001
**Malignancy (current+prior)**, n (%)	471/5929 (7.9)	178/2170 (8.2)	69/1223 (5.6)	0.002
**OSAS, n (%)**	254/5828 (4.4)	143/2105 (6.8)	47/1185 (4.0)	<0.001
**Hyperthyroidism**, n (%)	266/5856 (4.5)	99/2119 (4.7)	59/1205 (4.9)	0.860
**Hypothyroidism**, n (%)	511/5861 (8.7)	245/2122 (11.5)	117/1210 (9.7)	<0.001
**CKD**, n (%)	413/5917 (7.0)	408/2155 (18.9)	322/1230 (26.2)	<0.001
**CHA_2DS_2VASc**, median (IQR)	2 (1-4)	4 (3-5)	4 (3-5)	<0.001
**HASBLED**, median (IQR)	1 (1-2)	2 (1-2)	2 (1-3)	<0.001

**Devices, n (%)**	428/5849 (7.3)	219/2181 (10)	268/1212 (22.1)	<0.001
CRT-P/D, n (%)	18/428 (4.2)	20/219 (9.1)	86/268 (32.1)	
ICD, n (%)	72/428 (16.8)	28/219 (12.8)	113/268 (42.2)	
**Concomitant Drugs, n (%)**				
Any antiarrhythmic treatment	1752/5927 (29.6)	483/2176 (22.2)	290/1230 (23.6)	<0.001
ACE-inhibitors	2135/5929 (36.0)	999/2175 (45.9)	713/1233 (57.8)	<0.001
ARBs	1129/5926 (19.1)	511/2176 (23.5)	181/1233 (14.7)	<0.001
Beta-blockers	3855/5928 (65.0)	1612/2177 (74.0)	970/1232 (78.7)	<0.001
Aldosterone blockers	400/5926 (6.7)	610/2175 (28.0)	610/1232 (49.5)	<0.001
Diuretics	2140/5925 (36.1)	1512/2175 (69.5)	1032/1233 (83.7)	<0.001
Digoxin	522/5926 (8.8)	434/2176 (19.9)	391/1232 (31.7)	<0.001
Calcium channel blockers	1068/5925 (18.0)	388/2178 (17.8)	111/1233 (9.0)	<0.001
Non-DHP - CCB	334/5927 (5.6)	119/2178 (5.5)	50/1233 (4.1)	0.081
Statins	2309/5927 (39.0)	1024/2176 (47.1)	610/1232 (49.5)	<0.001
Oral antidiabetics	765/5929 (12.9)	418/2179 (19.2)	249/1233 (20.2)	<0.001
Insulin	215/5926 (3.6)	178/2178 (8.2)	116/1233 (9.4)	<0.001
**Antithrombotic treatment, n (%)**				<0.001
None	502/5948 (8.4)	100/2182 (4.6)	56/1235 (4.5)	
Only antiplatelets	343/5948 (5.8)	189/2182 (8.7)	93/1235 (7.5)	
VKA	2273/5948 (38.2)	1038/2182 (47.6)	543/1235 (44.0)	
NOACs	2250/5948 (37.8)	588/2182 (26.9)	276/1235 (22.3)	
Any OAC + Antiplatelets	580/5948 (9.8)	267/2182 (12.2)	267/1235 (21.6)	
**Optimal Medical Therapy, n (%)**	2278/5953 (38.3)	1218/2185 (55.7)	807/1235 (65.3)	<0.001
**ABC compliant, n (%)** *6027*	1386/4070 (34.1)	286/1306 (21.9)	182/651 (28.0)	<0.001

Legend: ABC: Atrial fibrillation Better Care; ACE, angiotensin converting enzyme; AF= atrial fibrillation; ARB, angiotensin receptor

blocker; BMI= body mass index; CABG= coronary artery bypass grafting; CAD= coronary artery disease; CCB= calcium channel blockers; CKD= chronic kidney disease; CMP= cardiomyopathy; COPD=chronic obstructive pulmonary disease; CV= cardiovascular; EHRA= European Heart Rate Association; DHP= Dihydropyridine; IQR= interquartile range; LVEF= left ventricular ejection fraction; MRAs= Mineralcorticoid Receptor Antagonists; NYHA=New York Heart Association PCI= percutaneous coronary intervention; NOAC= Non-Vitamin K Oral Anticoagulants; OAC= oral anticoagulants; PCI= percutaneous coronary intervention; TE= thromboembolic; TIA= transient ischaemic attack; VKA= Vitamin K antagonist.

Table S3: Major Adverse Outcomes throughout Follow-Up Observation according to LVEF Classification

	**No HF**<br>**n= 5953**	**HFpEF**<br>**n= 1662**	**HFmrEF**<br>**n= 523**	**HFrEF**<br>**n= 1235**	p
**Composite Outcome, n (%)**	638/5953 (10.7)	316/1662 (19.0)	114/523 (21.8)	366/1235 (29.6)	**<0.001**
**All-Cause Death, n (%)**	351/5944 (5.9)	222/1659 (13.4)	82/521 (15.7)	263/1223 (21.5)	**<0.001**
**MACEs, n (%)**	384/5951 (6.5)	197/1662 (11.9)	66/523 (12.6)	267/1235 (21.6)	**<0.001**
	**No HF**<br>**n=5953**	**HF >40% LVEF**<br>**n=2185**		**HF ≤40% LVEF**<br>**n=1235**	**p**
**Composite Outcome, n (%)**	638/5953 (10.7)	430/2185 (19.7)		366/1235 (29.6)	**<0.001**
**All-Cause Death, n (%)**	351/5944 (5.9)	304/2180 (13.9)		263/1223 (21.5)	**<0.001**
**MACEs, n (%)**	384/5951 (6.5)	263/2185 (12.0)		267/1235 (21.6)	**<0.001**

Legend: HF: Heart failure; HFpEF: Heart failure with preserved ejection fraction; HFmrEF: Heart failure with mildly reduced ejection fraction; HFrEF: Heart failure with reduced ejection fraction; MACE: Major Adverse Cardiovascular Event

Table S4: Major Adverse Outcomes throughout Follow-Up Observation according to LVEF Classification

	**HFpEF n= 1662**	**HFmrEF n= 523**	**HFrEF n= 1235**	p
**HF Worsening/Hospitalisation, n (%)**	101/1659 (6.1)	35/521 (6.7)	138/1223 (11.3)	**<0.001**
**HR (95% CI)***	Ref.	1.16 (0.77-1.76)	2.10 (1.57-2.82)	

	**HF >40% LVEF n=2185**	**HF ≤40% LVEF n=1235**	**p**
**HF Worsening/Hospitalisation, n (%)**	136/2180 (6.2)	138/1223 (11.3)	**<0.001**
**HR (95% CI)***	Ref.	2.01 (1.54-2.62)	

	**No OMT n= 1395**	**OMT n= 2025**	**p**
**HF Worsening/Hospitalisation, n (%)**	130/1391 (9.3)	144/2012 (7.2)	**0.025**
**OMT vs. No OMT, HR (95% CI)†**	Ref.	0.66 (0.51-0.86)	

	**No ABC n= 1489**	**ABC n= 468**	**p**
**HF Worsening/Hospitalisation, n (%)**	117/1486 (7.9)	42/468 (9.0)	**0.508**
**ABC vs. No ABC, HR (95% CI)†**	Ref.	1.11 (0.75-1.63)	

Legend: *adjusted for age, sex, hypertension, diabetes mellitus, coronary artery disease, any thromboembolic event, peripheral arterial disease, history of malignancy, chronic kidney disease, chronic obstructive pulmonary disease, type of AF and use of OAC; †adjusted for age, sex, hypertension, diabetes mellitus, coronary artery disease, any thromboembolic event, peripheral arterial disease, history of malignancy, chronic kidney disease, chronic obstructive pulmonary disease, type of HF (HFrEF, HFmrEF or HFpEF) and type of AF. ABC: Atrial fibrillation Better Care; OMT: Optimal Medical Therapy

Figure S1: Kaplan-Meier Curves for Composite Outcome according to LVEF Level

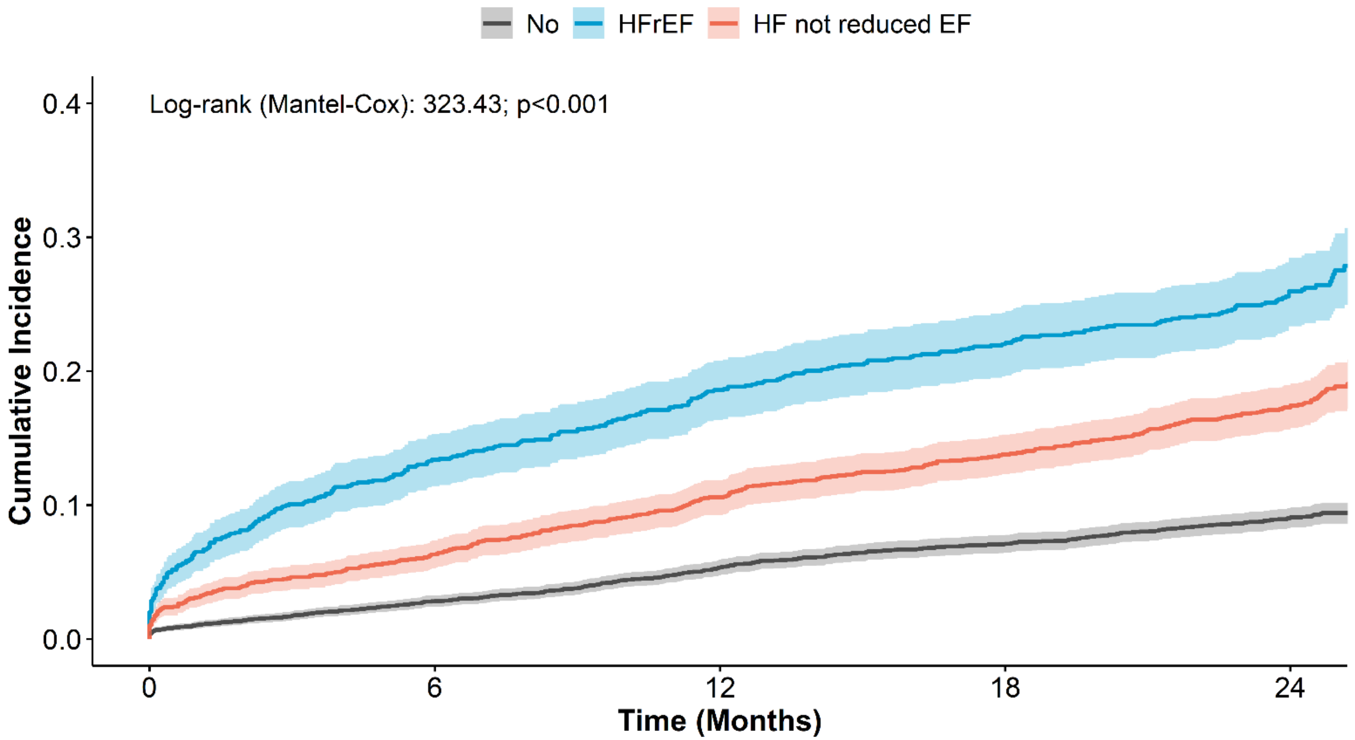

Number at risk

	0	6	12	18	24
No	5855	5681	5437	5033	3660
HFrEF	1181	1022	942	823	605
HF not reduced EF	2140	2001	1883	1687	1252

Time (Months)

Legend: HFrEF: Heart failure with reduced ejection fraction.

Figure S2: Regression Curve Analysis about LVEF and Risk of Composite Outcome in Overall and HF Patients

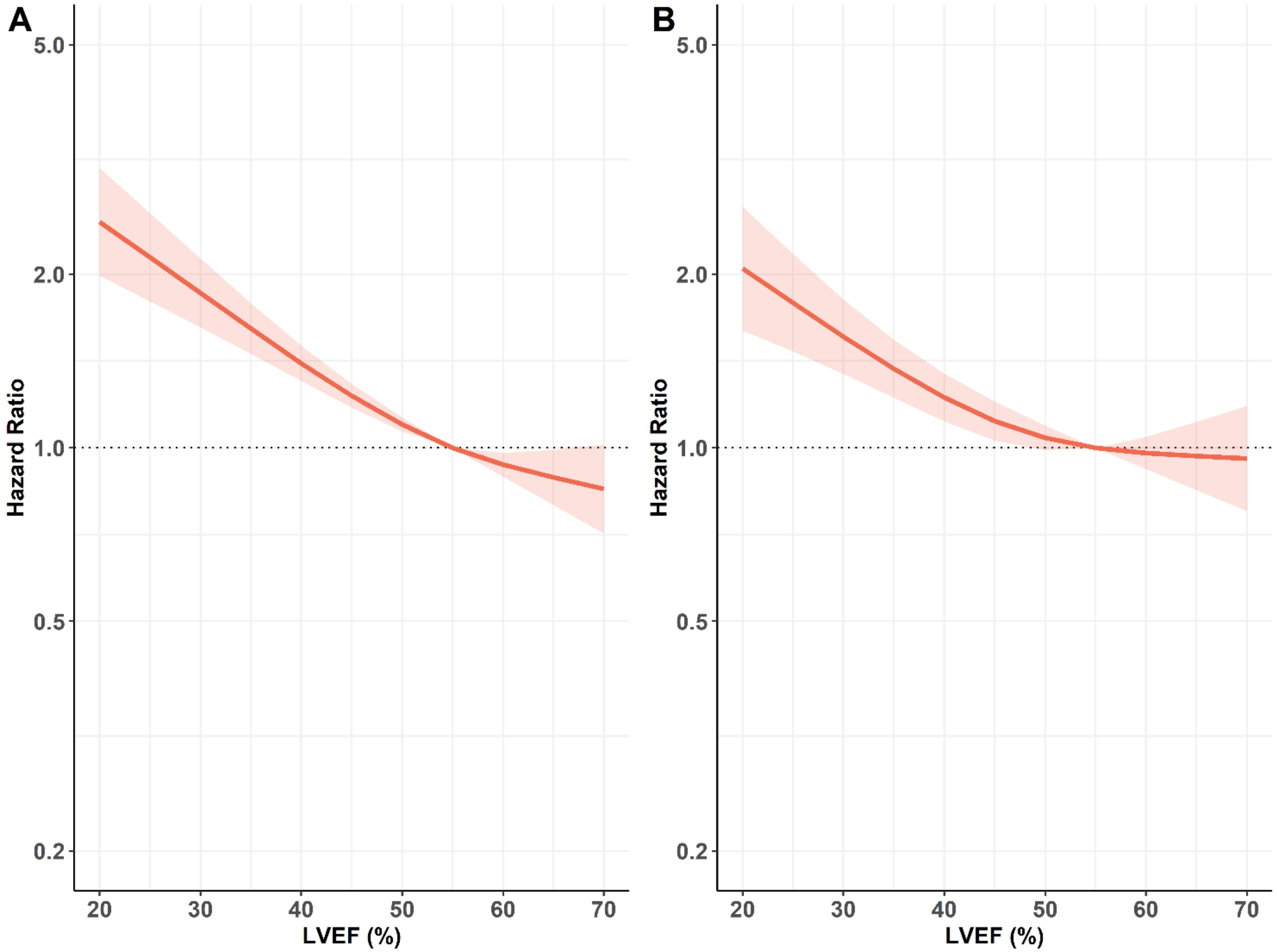

Legend: Panel A) Overall Cohort; Panel B) Patients with Heart Failure; LVEF= Left Ventricular Ejection Fraction.

Figure S3: Kaplan-Meier Curves for Composite Outcome according to Compliance to Optimal Medical Therapy in HF Patients

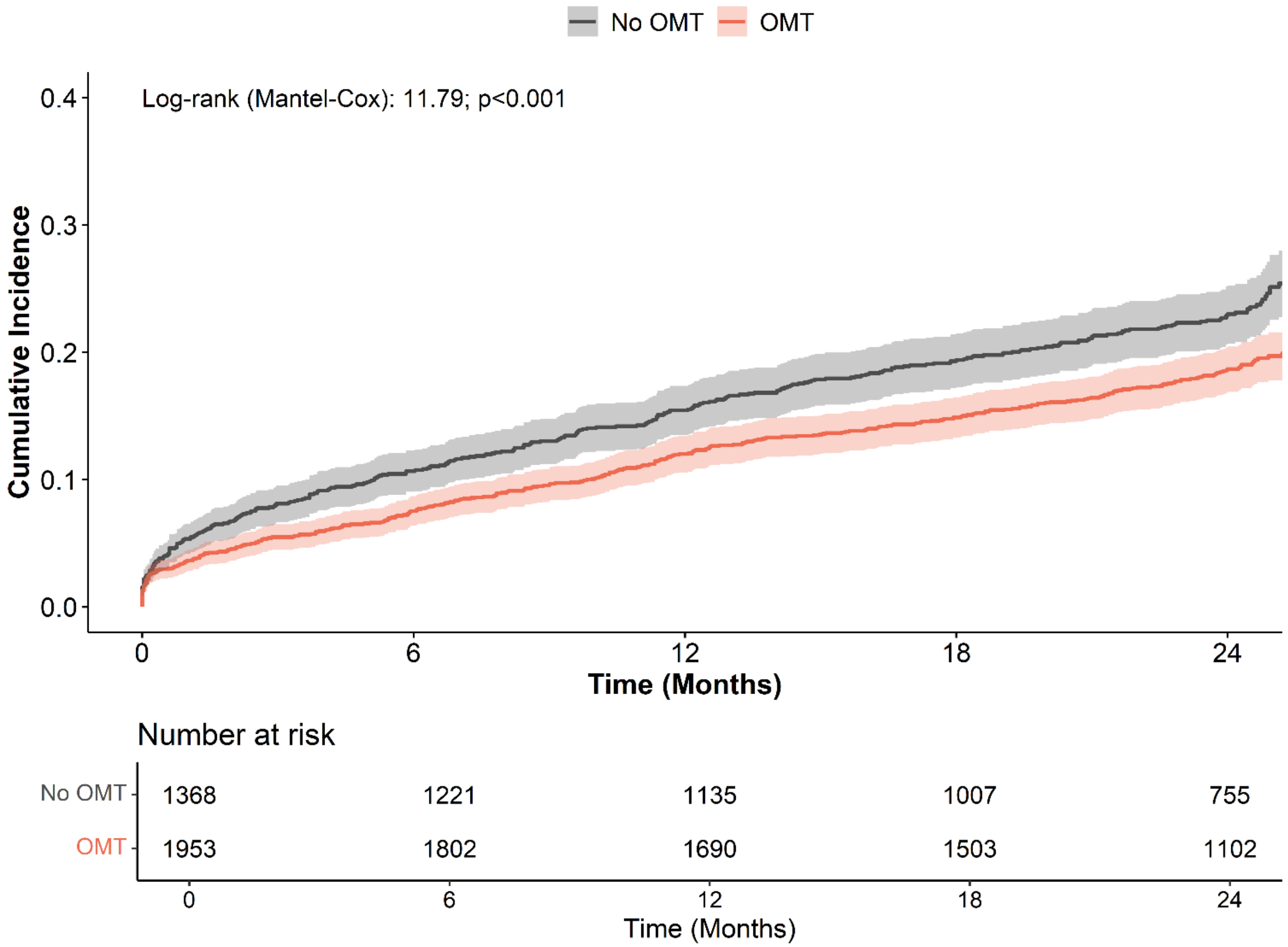

Legend: OMT: Optimal Medical Therapy

Figure S4: Kaplan-Meier Curves for Composite Outcome according to LVEF Level and Compliance to Optimal Medical Therapy.

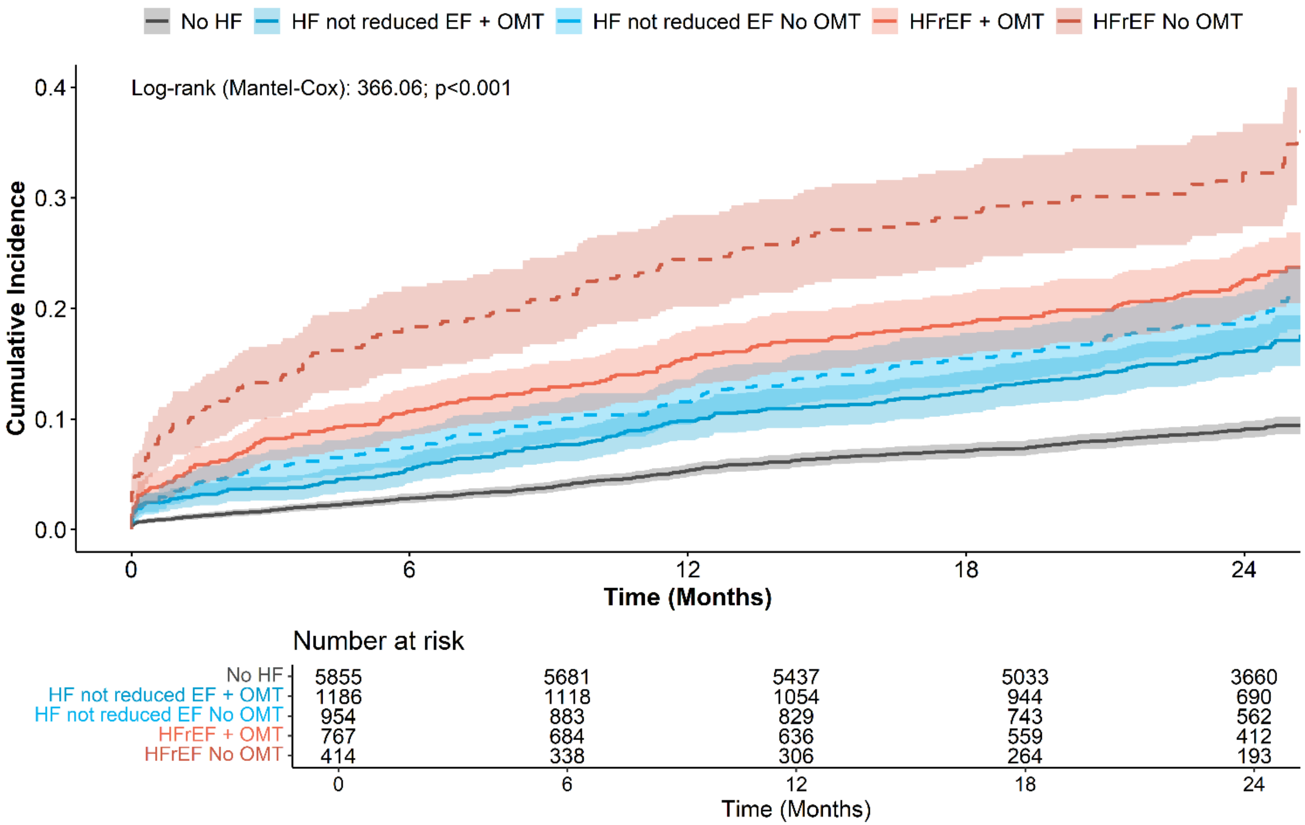

Legend: Black line: patients without HF (No HF); Blue solid line: HF patients with EF >40% (HF >40%) OMT compliant; Blue dashed line: HF >40%, no OMT compliant; Red solid line: HFrEF, OMT compliant; Red dashed line: HFrEF, no OMT compliant.

HF: Heart failure; HFpEF: Heart failure with preserved ejection fraction; HFmrEF: Heart failure with mildly reduced ejection fraction; HFrEF: Heart failure with reduced ejection fraction; OMT: Optimal Medical Therapy

Figure S5: Kaplan-Meier Curves for Composite Outcome according to LVEF Level and ABC Adherence

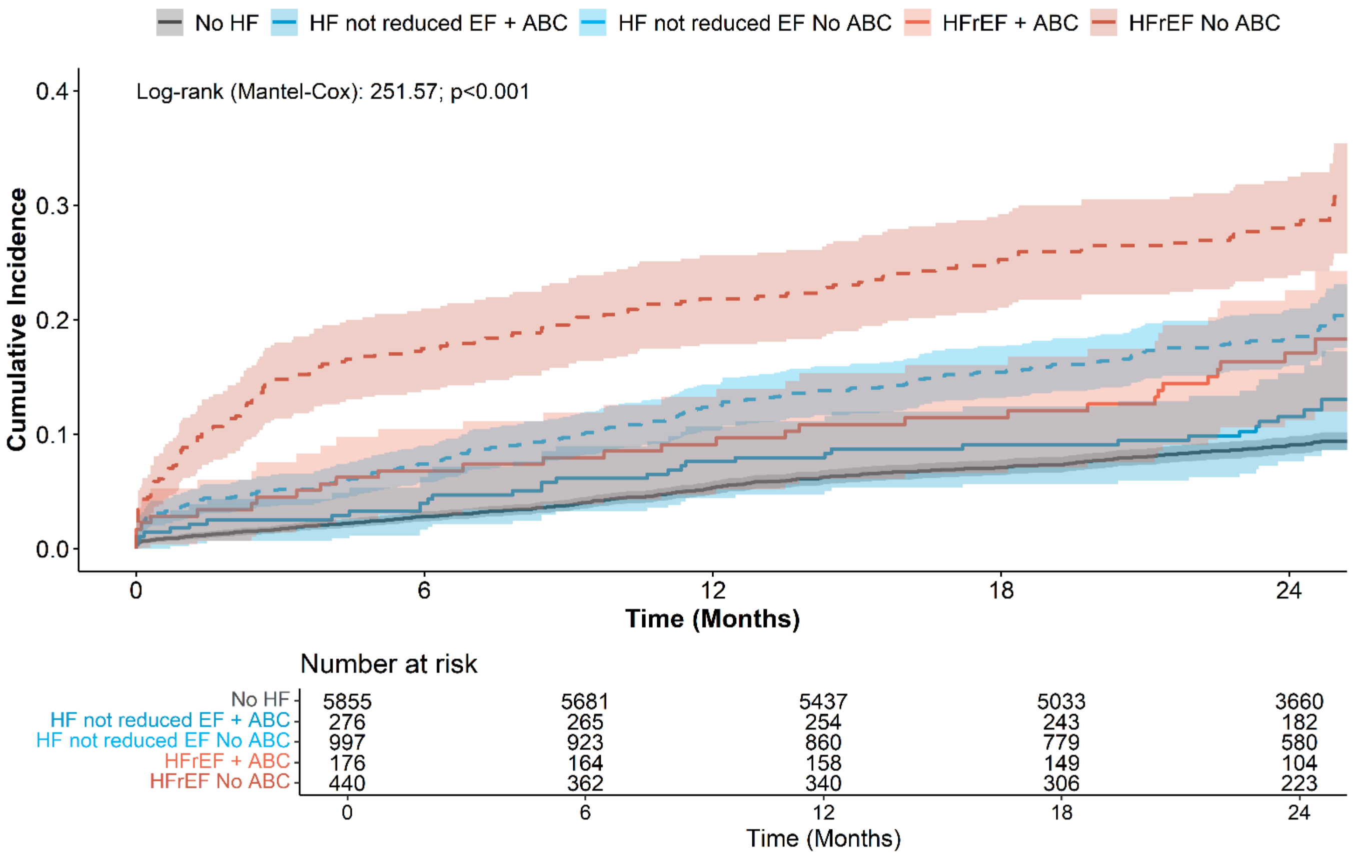

Legend: Black line: patients without HF (No HF); Blue solid line: HF patients with EF >40% (HF >40%), ABC compliant; Blue dashed line: HF >40%, no ABC compliant; Red solid line: HFrEF, ABC compliant; Red dashed line: HFrEF, no ABC compliant.

ABC: Atrial fibrillation Better Care; HF: Heart failure; HFpEF: Heart failure with preserved ejection fraction; HFmrEF: Heart failure with mildly reduced ejection fraction; HFrEF: Heart failure with reduced ejection fraction.
